# A computational study of Anthracyclines interacting with lipid bilayers: Correlation of membrane insertion rates, orientation effects and localisation with cytotoxicity

**DOI:** 10.1038/s41598-019-39411-y

**Published:** 2019-02-15

**Authors:** D. Toroz, I. R. Gould

**Affiliations:** Department of Chemistry, Imperial College London, London, SW7 2AZ UK

## Abstract

Anthracyclines interact with DNA and topoisomerase II as well as with cell membranes, and it is these latter interactions that can cause an increase in their cytotoxic activity. In the present study a detailed computational analysis of the initial insertion, orientation and nature of the interaction occurring between Anthracyclines and two different lipid bilayers (unsaturated POPC and saturated DMPC) is explored through molecular dynamics (MD) simulations; four Anthracyclines: Doxorubicin (DOX), Epirubicin (EPI), Idarubicin (IDA) and Daunorubicin (DAU) were examined. The results indicate that the increased cytotoxicity of DOX, in comparison to the other three analogues, is correlated with its ability to diffuse at a faster rate into the bilayers. Additionally, DOX exhibited considerably different orientational behaviour once incorporated into the bilayer and exhibited a higher propensity to interact with the hydrocarbon tails in both lipids indicating a higher probability of transport to the other leaflet of the bilayer.

## Introduction

**Figure 1:**
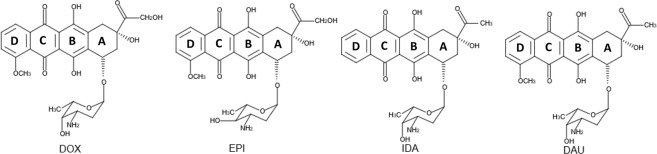
The four Anthracycline analogues Doxorubicin (DOX), Epirubicin (EPI), Idarubicin (IDA), Daunorubicin (DAU).

A significant problem in the treatment of cancer patients is the multidrug-resistance (MDR) phenotype which is associated with a decreased intracellular accumulation of the drug that appears to be mediated by the membrane glycoprotein, P-glycoprotein^1^. Various strategies have been applied to overcome MDR which include treatment of cells by using either chemosensitizers or antitumor drugs which can be concentrated into cells due to their specific molecular properties^2^; Anthracyclines, lipophilic antibiotics are one class of molecule which have been employed in clinical anticancer protocols^3^. The four commonly prescribed Anthracyclines are Doxorubicin (DOX), Epirubicin (EPI), Idarubicin (IDA), and Daunorubicin (DAU or DNR) which are illustrated in Fig. 1. EPI is a stereoisomer derivative of DOX which is characterised by exhibiting an increased volume of distribution and a longer half-life than DOX. IDA the derivative of DAU, which has had a methoxy group removed, exhibits an enhanced lipophilicity and higher cellular uptake than DAU. These drugs are known to intercalate into nucleic acids inhibiting DNA and nuclear RNA synthesis^4^. In addition, several studies have shown that the entry of Anthracyclines into tumour cells takes place via passive diffusion^5,6^. It has been suggested that the cell membrane is a target for drugs and more specifically the Anthracyclines can exert their cytotoxic activity solely through interaction with the cell membrane^7^. These observations prompted several researchers to perform studies to describe in detail the nature of the drug-lipid interactions^8–10^. However, it should also be noted that the hypothesis of direct membrane interaction is disputed in the literature especially at drug concentrations relevant to the typical therapeutic dose^11^.

Research has been conducted to assess the level of the acute toxicity of each of the four Anthracyclines, the order of acute toxicity of the four drugs found little correlation when assessed by *in vitro*, *in vivo* and clinical trials. The most toxic compound found from *in vitro*^12–18^ studies was IDA, with the most common ranking of acute toxicity being DOX<EPI<DAU<IDA. However, *in vivo*^12–22^ and clinical trials^23,24^ reported that the most toxic drug was DOX, with the most common order of acute toxicity being DAU<IDA<EPI<DOX. It has been demonstrated that the administration of the Anthracyclines leads to a marked inhibition of the membrane-associated calcium-independent phospholipase A_2_ (iPLA_2_). The rank order of toxicity found from the clinical studies was in correlation with a study performed on the four Anthracycline analogues for their ability to inhibit the Calcium-Independent Phospholipase A_2_ (iPLA_2_)^25^.

A recent computational study demonstrated the use of mechanistically interpretable molecular descriptors to identify the drug action of Anthracycline using quantum chemical calculations^26^. The contribution of these molecular descriptors to cytotoxicity were attested to as they can be interpreted as the intercalation between the quinone-containing tetracyclic rings of Anthracyclines and adjacent base pairs of DNA, and the passive diffusion of Anthracyclines through the lipid bilayers. A molecular level understanding of how the Anthracyclines interact with the lipid membrane, membrane insertion rate, orientation and ease of transport across the membrane, would be beneficial in the identification of molecular properties which could be applied in the design of more efficient compounds. Molecular dynamics simulations are a powerful tool as they can permit a detailed analysis of the contribution of the lipid components as well as the dynamics of the membrane bilayer when interacting with antitumor drugs. This manuscript reports the interactions occurring at the atomic level of detail between the four cancer therapeutic Anthracyclines with lipid bilayers and examines their behaviour in respect of their spontaneous membrane insertion, orientation and their location preferences when they are transported into a biological membrane environment. The issue of cell membrane inhomogeneity, the exact composition of the outer and inner leaflets in cell membranes being not quantitatively resolved, is addressed through the investigation of the Anthracyclines in two lipid bilayers with different characteristics (unsaturated and saturated hydrocarbon tails); this issue is frequently ignored in the majority of MD simulations of drug - lipid membrane systems. This manuscript reports on three key parameters (a) the initial membrane insertion times of the four analogues i.e. how fast each of the analogues enters the lipid bilayer, (b) the orientational position they prefer to retain in respect of the Z-direction of the lipid bilayer once inserted and (c) their location preferences into the different components of the lipids.

## Results

### Membrane insertion preferences of Anthracyclines into a lipid bilayer environment

To assess the “success” of membrane insertion of the analogues we have adapted the protocol described in Tian *et al*.^27^. The rate k_in_, the rate of analogue insertion, is related to the water/membrane partition coefficient via:

$$\begin{array}{c}{\bf{Drug}}+{\bf{membrane}}\underset{\mathop{\longrightarrow }\limits_{{{\boldsymbol{K}}}_{{\boldsymbol{out}}}}}{\overset{{\boldsymbol{Kin}}\,}{\longleftarrow }}{\bf{Drug}}+{\bf{membrane}}\\ {{\boldsymbol{K}}}_{{\boldsymbol{mem}}}=\frac{{{\boldsymbol{K}}}_{{\boldsymbol{in}}}}{{{\boldsymbol{K}}}_{{\boldsymbol{out}}}}\end{array}$$

Whilst we cannot directly calculate K_mem_ since we do not have access to K_out_ data, we are able to assess K_in_ and use this as a metric to quantify the membrane insertion propensity of the analogues. From Tian *et al*.^27^ the association constant K_in_ is calculated from the equation

$${{\boldsymbol{K}}}_{{\boldsymbol{in}}}=\frac{{\boldsymbol{A}}{{\boldsymbol{L}}}_{{\boldsymbol{z}}}}{ < {\boldsymbol{W}} > }$$

where A is the size of the phospholipid surface and L_z_ is the thickness of the water layer that the analogue diffuses in to the bilayer and <W> is the mean first passage time that each of the analogues reaches the surface of the lipid. Table 1 represents the association constants of each of the anthracycline analogues in the two lipids.

**Table 1:** Association constants K_in_ of the four anthracyclines in the POPC and DMPC lipids (units in M^−1^ S^−1^).

Lipid	DOX	EPI	IDA	DAU
POPC	3.71 × 10^9^	2.75 × 10^9^	3.09 × 10^9^	3.42 × 10^9^
DMPC	2.00 × 10^9^	1.40 × 10^9^	1.50 × 10^9^	1.88 × 10^9^

From Table 1 we note that the values of the K_in_ are of a similar magnitude as that obtained for the di-mannose investigation of Tian *et al*.^27^, where they obtained a value from simulation of 5.15 × 10^9^ M^−1^ S^−1^, indicating that our simulations are reporting similar timescales. Examining first the POPC results, DOX has the highest K_in_ value followed by DAU then IDA and finally EPI, this yields an order for successful membrane insertion of DOX>DAU>IDA>EPI. Considering the magnitude of the K_in_ values, DAU’s value is within 10% of DOX’s indicating that it would insert into the bilayer with a similar rate of success. IDA’s value is within 20% of DOX whilst EPI’s association constant, K_in_ of 2.75 × 10^9^ is noticeably slower, by 25%, than DOX indicating it would be significantly less successful in inserting into the bilayer. The order of membrane insertion for the DMPC bilayer is the same as for POPC with DOX>DAU>IDA>EPI, indicating that the nature of the bilayer does not alter relative propensity of the analogues to insert into the bilayer.

However, what is significant in the results of DMPC is the observation that the values of K_in_ are approximately half the value of those observed in POPC. This behaviour can be rationalised in respect of the lipid tails of POPC and DMPC, since both have the same phosphocholine head group, the former is composed of a saturated Palmitoyl and an unsaturated Oleoyl tail whilst the latter is composed of only saturated Myristoyl tails. For POPC the presence of the unsaturated Oleoyl tail results in significantly reduced order parameters for both tail’s and consequently larger area per head group values, therefore, the structure of the bilayer is more fluid than DMPC, this being reflected in the transition temperature of 271.15 K for POPC and 297.15 K for DMPC. It is worth reiterating that whilst the K_in_ values in DMPC are smaller than those in POPC they exhibit the same order for Anthracycline membrane insertion. In the Supplementary Material we provide videos illustrating the insertion process of the Anthracyclines in both POPC and DMPC, it can be observed in several of the videos that the particular analogue is completely free to rotate prior to insertion.

### Orientation preferences of Anthracyclines into a lipid bilayer environment

**Figure 2:**
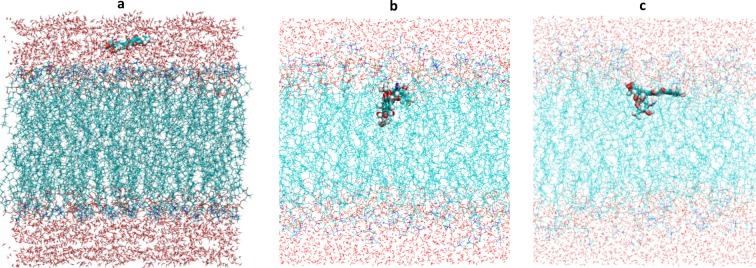
(**a**) Initial position of the Anthracycline-lipid system at the start of the simulations. Definition of orientation the Anthracyclines prefer when diffused into the bilayers (**b**) Perpendicular orientation of Anthracycline (tetracyclic ring containing adjacent quinone-hydroquinone groups) to the bilayer (**c**) Horizontal orientation (tetracyclic ring containing adjacent quinone-hydroquinone groups) of Anthracycline to the bilayer.

Following investigation of the permeability properties of the Anthracyclines analogues, the orientational behaviour of the analogues once inserted into the lipid bilayers were examined. The criteria used to monitor the orientational preferences were the orientation of the Daunosamine, the phenyl ring of the cyclic group (anthraquinone residue) (labelled D) and the Tetracyclic ring (labelled A) with respect to the z-dimension of the lipid bilayer, Fig. 2b,c. The angle of orientation was computed for all the repeats from the moment that each of the analogues were inserted into the lipid bilayer until the end of the simulation sampling time. For Horizontal orientation the angle range considered around 180 or 0 degrees (+/−10 degrees) whereas for the Perpendicular orientation was considered around 90 degrees (+/−). Table 2 presents the orientation preferences of the four analogues in the two lipids. In the Supplementary Material we provide angle distribution data for 3 of the 12 repeats for each of the analogues in the two lipids, Figs 1Sa through 2Sc.

**Table 2:** Orientation preferences of Anthracyclines to POPC and DMPC lipid bilayers (angles in degrees).

Lipid Bilayer	POPC				DMPC			
Anthracycline analogue	DOX	EPI	IDA	DAU	DOX	EPI	IDA	DAU
Angle1-Daunosamine	H/P	H	H	H	H/P	H	H/P	H/P
Angle2-Phenyl Ring (D)	H/P	P	P	P	H/P	P	P	P
Angle3-Tetracyclic ring (A)	P	P	P	P	P	P	P	P

From the results obtained for the Daunosamine subunit, the orientation angle varies between the different analogues and presents different behaviour in the two lipids. In POPC the Daunosamine of DOX varies in position showing a preference to rotate whereas for the other three analogues a horizontal position for EPI, IDA and DAU is exhibited. In DMPC Daunosamine in DOX demonstrates the same behaviour as observed in POPC as does EPI. IDA and DAU exhibit behaviour similar to DOX in DMPC with both showing either a horizontal or perpendicular position, indicating their propensity to rotate in the bilayer. For the Anthraquinone ring (D**)** the orientational preferences are identical, in both lipids, for EPI, IDA and DAU all having an orientation perpendicular to the z-direction. DOX, as for the Daunosamine angle, in both lipids, exhibits no preference with it adopting both horizontal and perpendicular orientations; this emphasises that DOX is far more flexible in its orientation in the lipid bilayer. The Tetracyclic ring (A) retains a perpendicular position in respect to the z-dimension of the lipid bilayer for all the analogues and in both lipids. The conclusion that emerges from the results is that as the four analogues enter the bilayer the fused six-member ring retains a perpendicular orientation apart from DOX where it presents a bending angle on the last ring (D). Additionally, for the Daunosamine subunit, DOX retains a perpendicular orientation, EPI a bending angle orientation and IDA as well as DAU (more horizontal orientation in POPC and more perpendicular in DMPC) the orientation preference varies with the lipid into which they are diffused.

### Preference of location of Anthracyclines within the different lipid bilayers

**Figure 3:**
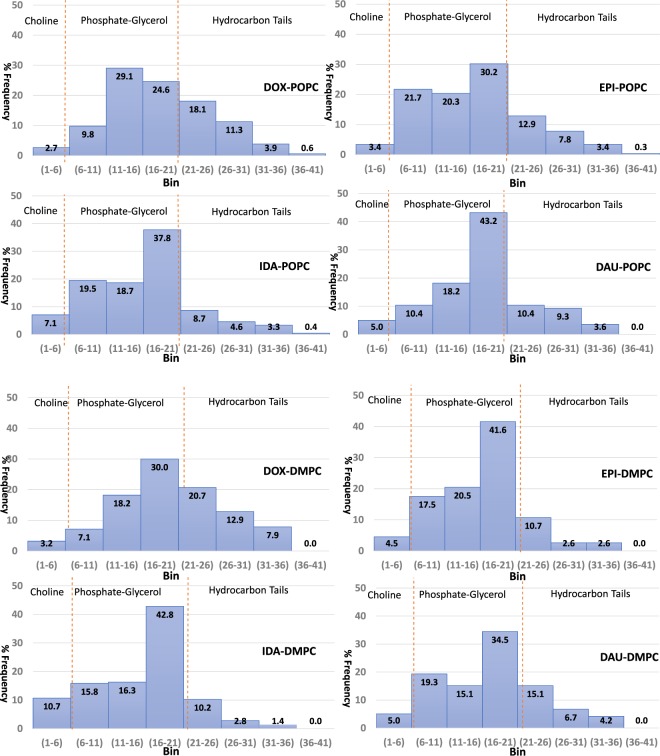
Preference of interactions between the four Anthracyclines during the simulation with the different regions of the two lipids. The x-axis represents the bin of the histograms whereas the y-axis represents as a percentage of the occurrence of each of the interactions of Anthracyclines with the specific region of the lipid components.

It is instructive to examine the location of the analogues within the bilayer once they have entered the membrane by characterising their interactions with either the head group, phosphate or tail regions. Figure 3 shows the propensity of the interactions of the four analogues with the different regions of the two different lipids. The choice of the bins that represent the atoms of the different components of the lipids are presented in the Supplementary Information File Fig. 3S).

**Figure 4:**
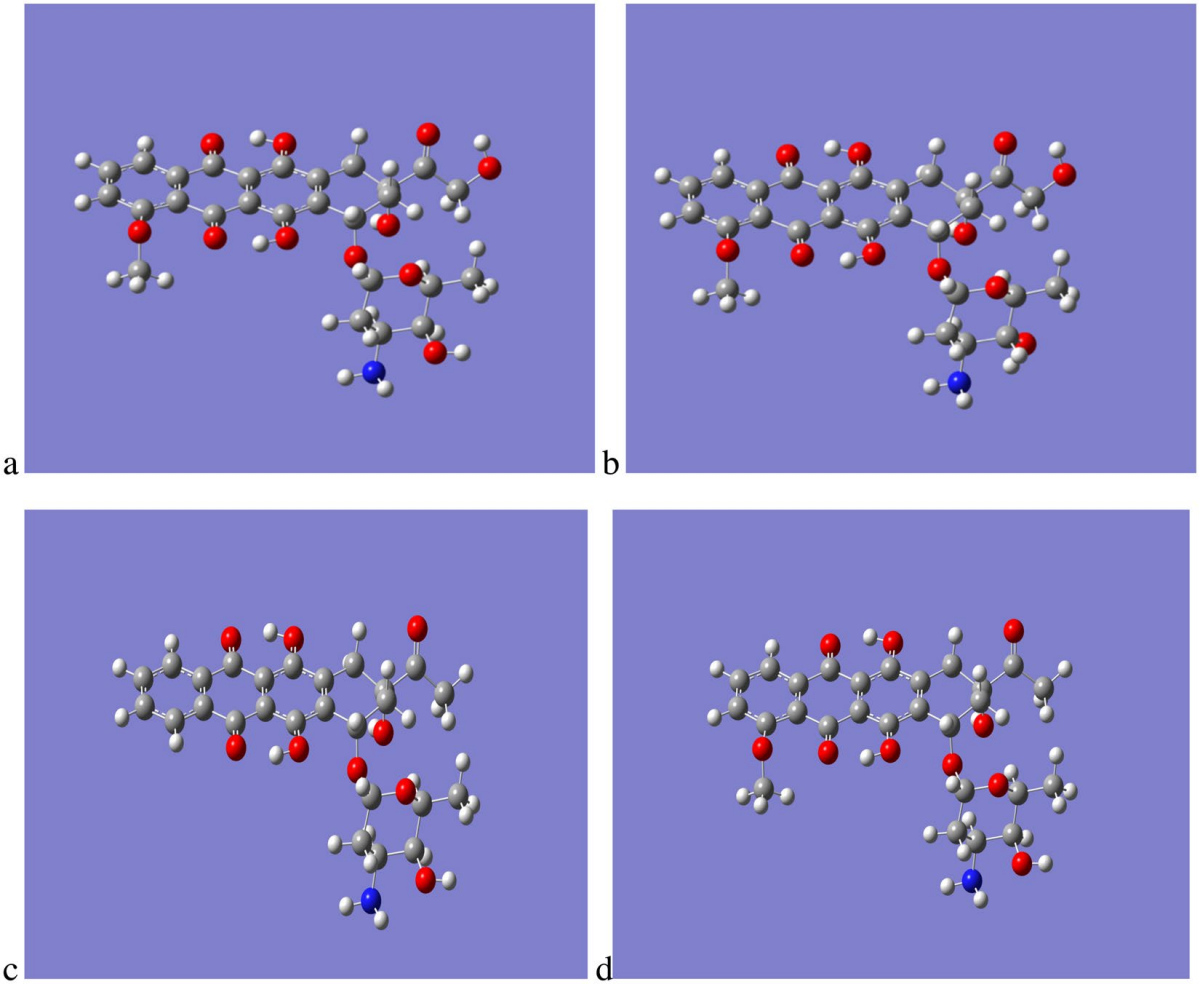
Optimised QM gas phase structures of (**a**) DOX, (**b**) EPI, (**c**) IDA and (**d**) DAU.

From Fig. 3 it is clearly identified that for all of the analogues, irrespective of lipid environment, they interact most frequently with the phosphate region. From the data there is a clear difference in the behaviour of DOX, compared to the other analogues, with it interacting far more frequently with the hydrocarbon tails, in both lipids. Therefore, DOX not only enters the bilayers the quickest but it also has the propensity to reach the hydrocarbon tails and will therefore have a higher probability to traverse to the other leaflet of the bilayer^27,28^. Examining EPI in the two lipids reveals that in POPC the behaviour is more similar to DOX in respect of EPI’s frequency of interactions with the lipid tails; in DMPC this behaviour is attenuated with a correspondingly higher frequency of interaction with the phosphate region. The sensitivity to the lipid environment in the localisation of DOX and EPI in the bilayers again is surprising given their chemical similarity. Considering their behaviour in POPC it is unsurprising that the distribution of interactions is somewhat similar, both penetrating into the tail region, though DOX having a higher frequency of interaction, as the Oleoyl tail of POPC has a low order parameter indicating enhanced flexibility and therefore a lower barrier to penetration. In DMPC the Myristoyl tails are shorter than both Oleoyl and Palmitoyl resulting in a reduced bilayer thickness, 35 Å DMPC vs 37 Å POPC, but as the tails are fully saturated the order parameters for the tails are higher than POPC indicating that they are more ordered and require more energy to penetrate. Therefore, DOX’s behaviour in DMPC indicates that its interactions are of a magnitude similar to that of the lipid-lipid tail interactions to facilitate its insertion, for EPI the interaction energy from the frequency of interactions with the tails indicates that it is not sufficient to overcome the tail-tail packing interaction. The behaviour of IDA in both lipids is very similar, exhibiting the lowest frequency of interaction with the tails compared with the other analogues, this is somewhat surprising as it is believed that IDA’s increased toxicity relative to DAU is due to its increased lipophilicity; the only significant difference that IDA displays relative to the other analogues is a higher frequency of interaction with the, Choline, head group. In comparison to IDA, DAU’s behaviour in both lipids clearly demonstrates a high propensity to migrate to the tails of the lipids, this behaviour is quite similar to that of DOX, the major difference between the two being speed and relative success of penetration with DOX being much quicker and more likely to penetrate the membrane that DAU. As DOX inserts much faster than the rest of the analogues and interacts more with the hydrocarbon tails we have performed the same analysis for the first 60 ns of the simulations to ensure that these results are not due to the membrane insertion rates observed. The results are presented in the electronic Supplementary Information File (Fig. 4S) and show similar behaviour as the results obtained from the 100 ns simulations.

### Electronic and Vibrational differences of DOX and EPI

We have performed high quality density functional calculations on DOX and EPI to investigate the differences in their physical properties in order to rationalise their markedly different behaviour in respect to their membrane insertion properties given that they are simply stereoisomers of each other; for completeness we have also performed the same analysis for IDA and DAU. From the gas phase optimisations EPI is identified as the lowest energy isomer, being more stable than DOX by 12 KJ/Mol. The gas phase optimized structures of DOX and EPI are shown in Fig. 4, along with those of IDA and DAU. Unsurprisingly there is a significant degree of overlap in the structures of DOX and EPI with the only real difference occurring in the sugar Duanosamine, for DOX the OH is orientated such that it forms an intra-molecular hydrogen bond between the Oxygen of the OH, therefore acting as a H-bond acceptor, and an amine Hydrogen, acting as an H-bond donor; the intra-molecular H-bond having a distance of 2.3 Å. In contrast, for EPI the OH is orientated such that it acts as an H-bond donor to the N of the amine group, with an intra-molecular H-bond with a distance 2.2 Å; PDB’s of the optimised structures are provided in the Supplementary Material.

**Figure 5:**
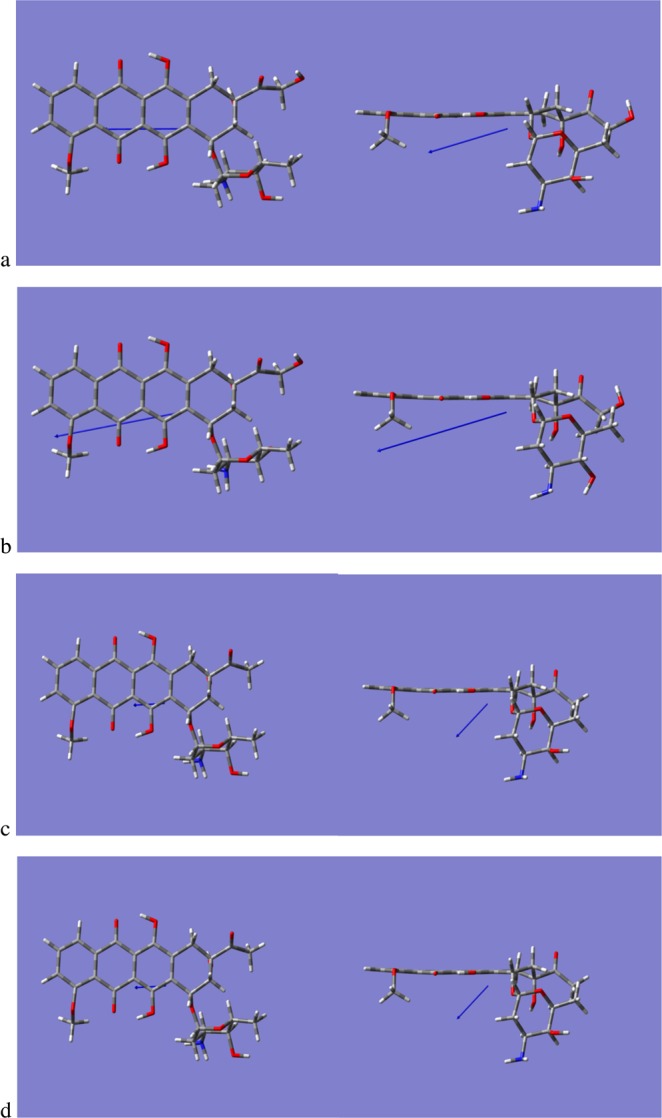
Dipole Moments of (**a**) DOX, (**b**) EPI, (**c**) IDA and DAU (**d**).

In Fig. 5 we show the magnitude and direction of the dipole moments for the Antrhacyclines, it is clear that they all exhibit the same directionality. However, there are significant differences in the magnitude of the dipole moments with EPI having a dipole moment of 7.5 Debyes (D) and 4.5D respectively. For IDA and DAU the dipole moments are of smaller magnitude at 2.15 D and 2.83 D respectively. For molecules with large dipole moments there will be stronger aqueous solvation due to the preferential interaction with the dipole moments of the surrounding water molecules, this would indicate that EPI would have a more favourable solvation free energy in water than DOX and therefore would be more stable in the water phase.

**Figure 6:**
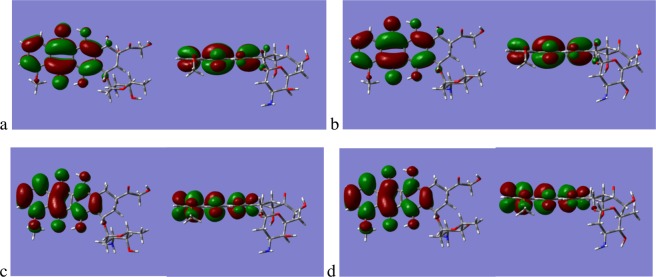
(**a**) LUMO, MO = 144, of DOX, (**b**) LUMO of EPI, (**c**) LUMO + 1, MO=145, of DOX and (**d**) LUMO + 1 of EPI.

**Figure 7:**
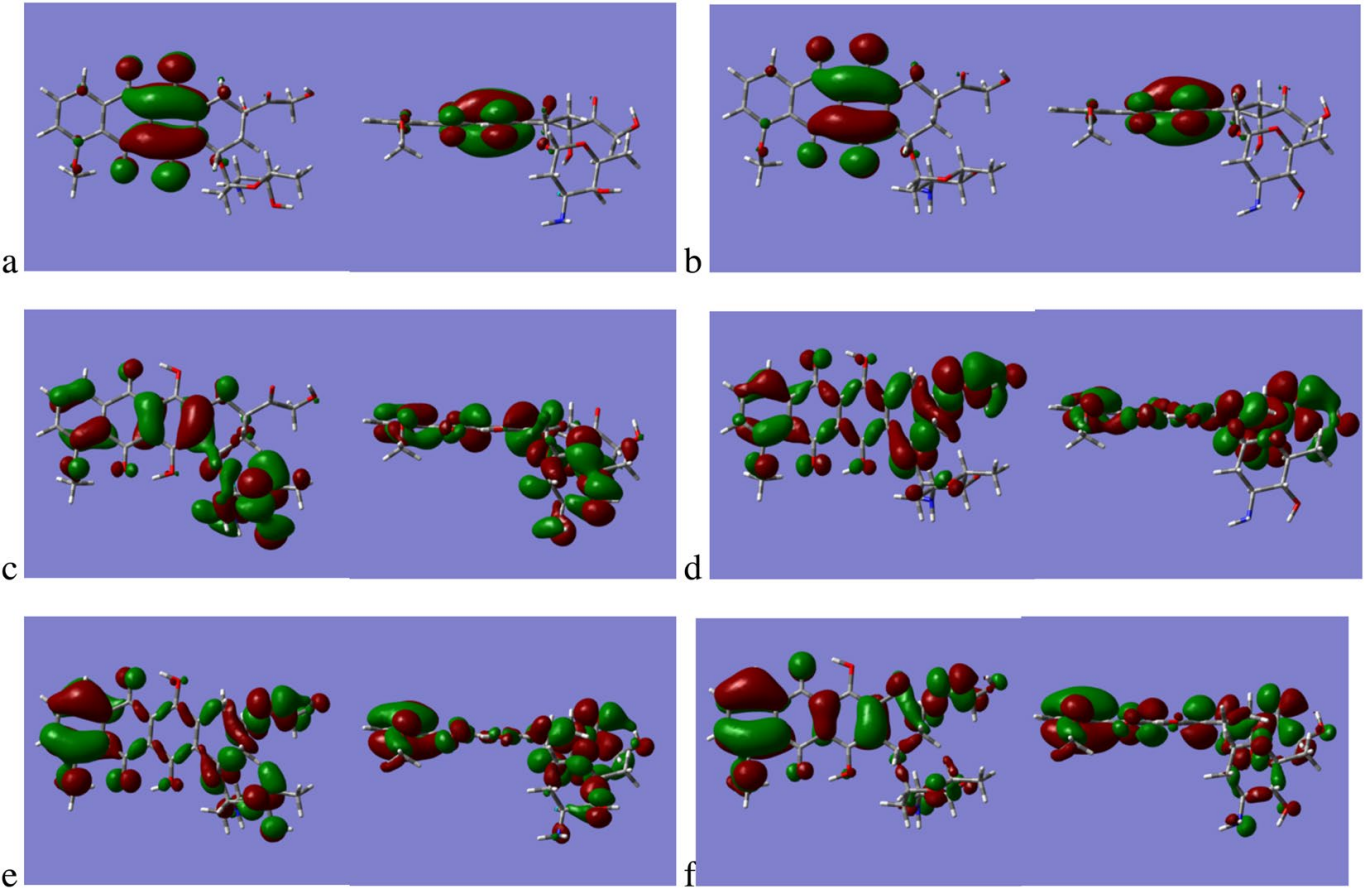
(**a**) HOMO, MO = 143, of DOX (**b**) HOMO of EPI, (**c**) HOMO-2, MO = 141, of DOX, (**d**) HOMO-2 of EPI, (**e**) HOMO-3, MO = 140, of DOX and (**f**) HOMO-3 of EPI.

Expanding upon the dipole moment analysis it is also instructive to examine the differences in the occupied and unoccupied molecular orbitals (MO’s) for DOX and EPI, as they are stereoisomers the total number of MO’s is the same and the Highest Occupied Molecular Orbital (HOMO) is located at MO = 143 and the Lowest Unoccupied Molecular Orbital (LUMO) at MO = 144. Examination of the LUMO, and the four unoccupied MO’s above it, illustrate that there are no significant differences in the form of the orbitals, Fig. 6 illustrates the LUMO and the LUMO + 1 for DOX and EPI. In the Supplementary Material, Fig. S5, we show the MO’s for both DOX and EPI from MO = 135 through MO = 152, that is the 10 highest occupied and the 10 lowest unoccupied orbitals. The picture for the occupied MO’s is very different to the unoccupied orbitals, with the exception of the HOMO and the HOMO-1 which are very similar between DOX and EPI. In Fig. 7 we show the HOMO for DOX and EPI for reference and the HOMO-2, HOMO-3, the reader is directed to the Supplementary Data in Fig. 5S for the other MO’s. For the HOMO-2 and HOMO-3 of both DOX and EPI the orbitals are spread across the ring backbone in a similar manner, the most obvious difference for HOMO-2 being that the orbital is located on the sugar Duanosamine for DOX but is located on the carbonyl and alcohol units of the Phenyl ring for EPI. The HOMO-3 orbital in the case of DOX appears approximately equally distributed on the Duanosamine and the carbonyl and alcohol, whilst for EPI it is distributed more on the carbonyl and alcohol with no presence on the Duanosamine. The differences in the occupied MO’s of the two molecules indicates the potential origin of the difference in the magnitude of their dipole moments, and also suggests that the interaction between the Anthracyclines and the lipids may be the result of the ability to donate electron density from the Anthracycline occupied MO’s.

**Figure 8:**
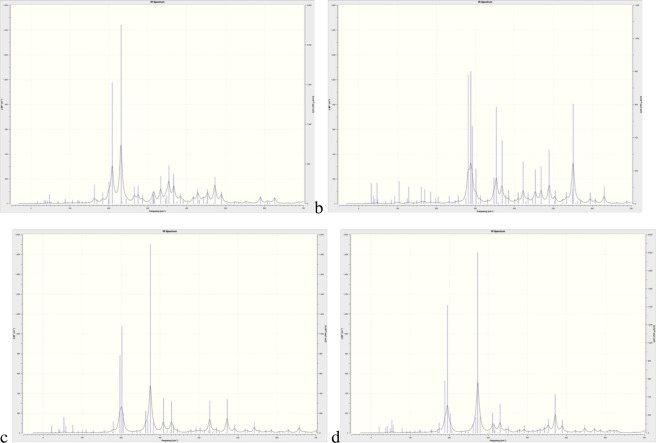
Calculated IR spectrum in the spectral window 0–600 cm^−1^ for (**a**) DOX, (**b**) EPI, (**c**) IDA and (**d**) DAU.

Identification that the optimised QM structures were true local minima was confirmed by calculation of the vibrational frequencies for the Anthracyclines, all structures exhibitied no imaginary frequencies confirming that they were stationary states. The vibrational frequency analysis revealed clear differences in the behaviour of DOX and EPI in the sub 600 cm^−1^ region of the spectra as illustrated in Fig. 8; the spectra in this region for IDA and DAU was similar to that of DOX. The full calculated spectra for all of the Anthracyclines is available in the Supplementary Material Fig. 6S.

**Figure 9:**
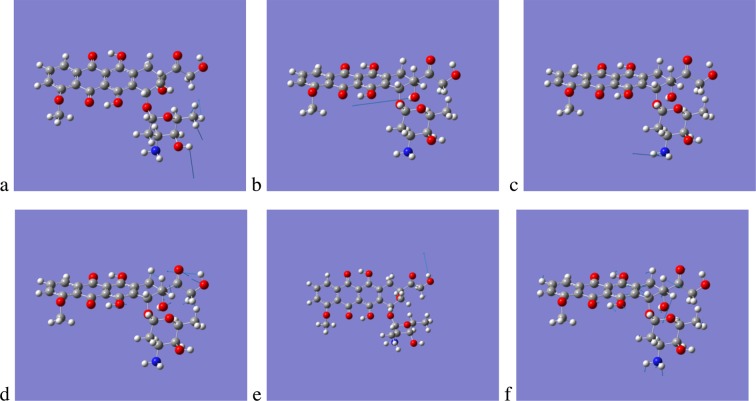
Active vibrational modes with displacement vectors for DOX in the 0–600 cm^−1^ region (**a**) 209 cm^−1^, (**b**) 231 cm^−1^, (**c**) 333 cm^−1^, (**d**) 354 cm^−1^, (**e**) 367 cm^−1^ and (**f**) 472 cm^−1^. Arrows represent magnitude and direction of the displacements.

From Fig. 8 an explanation for the difference in the behaviour of DOX and EPI with regards to their capability to achieve membrane insertion can be derived from examination of the low- lying vibrational modes, their nature and magnitude of absorption. Considering only vibrations with absorbance strength epsilon, ε, greater than 200 M^−1^ cm^−1^, there are only 6 active modes for DOX in the region 0–600 cm^−1^, illustrated in Fig. 9; animations of the vibrational modes can be found in the Supplementary Material.

The ratio of the calculated absorbances for the 6 frequencies of DOX, 209 through 472 cm^−1^ in Fig. 9 is 6:10:2:3:2:3 respectively. The first two frequencies have the largest magnitudes of absorption and are both very localised in respect to the structure of DOX, vibrational frequency 209 cm^−1^ is related to the rocking motion of the methyl group and the rotation of the hydroxyl group both on the sugar Duanosamine. The 231 cm^−1^ vibration, the strongest absorption below 600 cm^−1^, is associated with the rotation of the Hydrogen of the hydroxyl group attached to the tetracyclic ring A with the second strongest absorption at 209 cm^−1^ being a combination of the Daunosamine methyl group rotation and the Hydrogen rotation of the hydroxyl group. Examination of all six vibrations shows that they are very localised in their displacements, videos of all six vibrations are available in the Supplementary Material, indicating that the rings (A–D) for DOX are rigid.

Examination of the vibrations for EPI in the region below 600 cm^−1^ reveals that there are 12 vibrational modes whose absorbance ε is greater than 200 M^−1^ cm^−1^ double the number of DOX.

**Figure 10:**
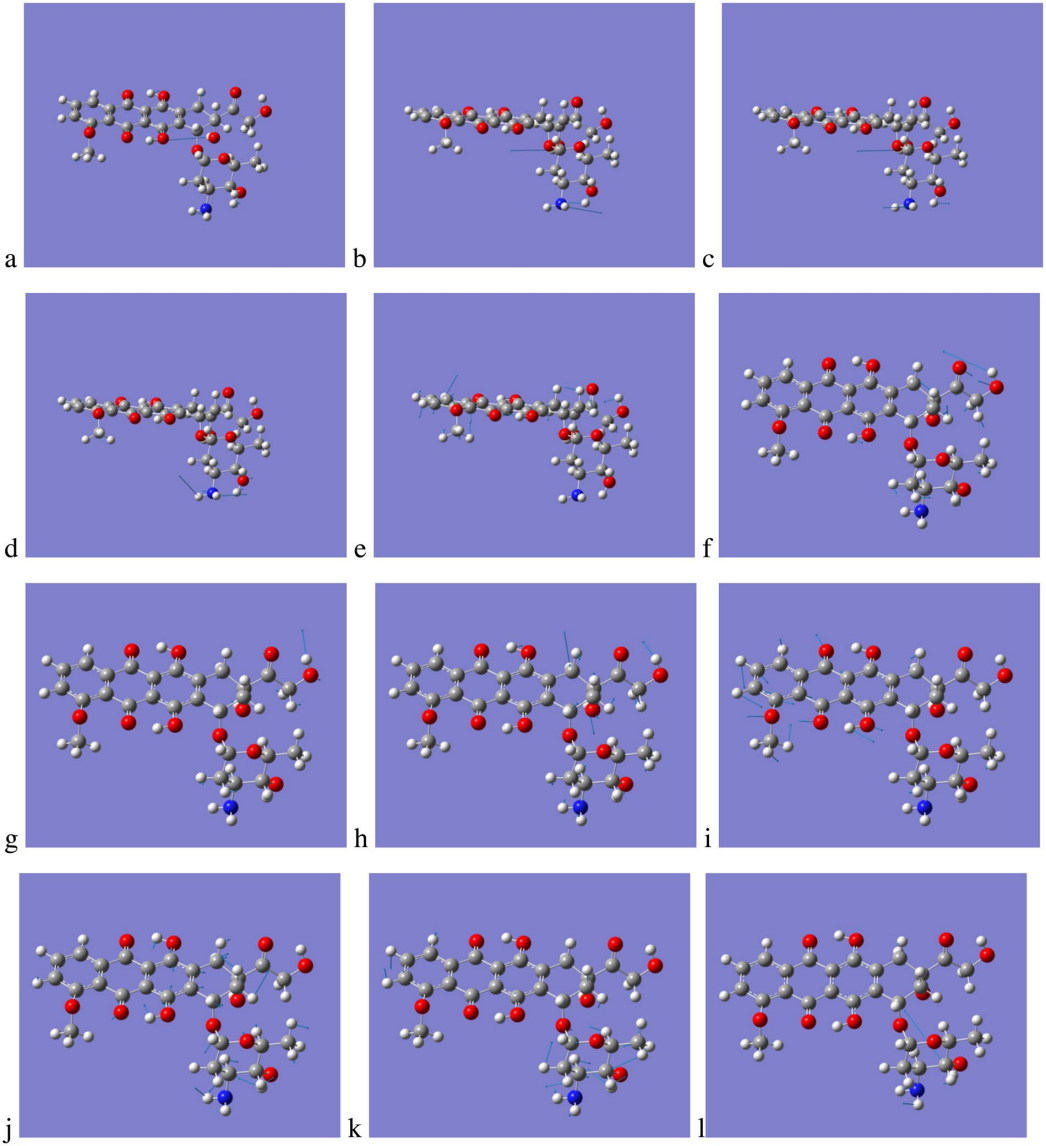
Active vibrational modes with displacement vectors for EPI in the 0–600 cm^−1^ region (**a**) 281 cm^−1^, (**b**) 287 cm^−1^, (**c**) 292 cm^−1^, (**d**) 302 cm^−1^, (**e**) 347 cm^−1^, (**f**) 353 cm^−1^, (**g**) 368 cm^−1^, (**h**) 422 cm^−1^, (**i**) 453 cm^−1^, (**j**) 468 cm^−1^, (**k**) 489 cm^−1^ and (**l**) 552 cm^−1^. Arrows represent magnitude and direction of the displacements.

As for DOX videos of all twelve vibrations are available in the Supplementary Material, the ratio, to the nearest integer, of the calculated absorbances for the 12 frequencies of EPI, 281 through 552 cm^−1^ in Fig. 10 is 4:4:3:1:1:4:3:2:2:2:3:4 respectively. Analysis of the first three vibrational frequencies, 281, 287 and 292 cm^−1^, reveals different behaviour when compared with the first two frequencies of DOX, all three involve the rotation of the Tetracylic A ring hydroxyl coupled with motion of the amine and hydroxyl group of the Daunosamine, furthermore, for the vibration at 292 cm^−1^ there is also in- and out- of plane distortion of the rings D through A. The vibration at 347 cm^−1^ though relatively weak exhibits marked out of plane deformation for rings C and D along with distortion of the carbonyl and alcohol attached to the Tetracylic ring A. There is a strongly coupled vibration between the carbonyl and alcohol of the Tetracylic ring A at 353 cm^−1^ along with in-plane motion of the rings B through D and significant motion of the Daunosamine. The vibration at 368 cm^−1^ is mostly comprised of the rotation of the alcohol hydrogen, but there is also motion associated with out of plane distortion of the rings B through D. The vibrational frequencies 422, 453 and 468 cm^−1^ all have similar strengths of absorption, the first of these vibrations corresponds to an extremely coupled rocking mode between the CH_2_ and hydroxyl group adjacent to each other on the tetracyclic ring A and also rocking of the alcohol OH; there is also motion associated with the Duanosamine ring. The next vibration at 453 cm^−1^ is associated with large in plane and smaller out of plane deformations of the ring B through D, whilst the mode at 468 cm^−1^ shows ring bending motion of the Duanosamine, distortion of the tetracyclic ring A and in plane stretching and bending of the rings B through D. The relatively strong vibration at 489 cm^−1^ is particularly noteworthy as it exhibits a strong absorption and is composed of Daunosamine distortion and also significantly out of plane distortion of the rings D through A.

Comparing the low frequency IR modes for DOX and EPI, clearly identifies that DOX is far more rigid than EPI, which has double the number of significant vibrational modes in the 0–600 cm^−1^. Examination of the same spectral region for IDA and DAU reveals that they have 9 and 7 significant vibrational modes respectively. This suggests that there is a correlation between the rigidity of the Anthracycline and its ability to insert into the lipid bilayer as the number of significant vibrational modes DOX (6), DAU (7), IDA (9) and EPI (12) is in the same order as the rank order of insertion DOX<DAU~IDA<EPI, values in parenthesis are the number of vibrational modes in the region 0–600 cm^−1^ which have absorbance, ε, greater than 200 M^−1^ cm^−1^.

## Discussion and Conclusions

This work has demonstrated that for the four Anthracyclines studied DOX consistently had the highest success of membrane insertion and the deepest penetration into both POPC and DMPC bilayers suggesting that its cytotoxicity can be correlated with these parameters. The substantial differences observed in the measurements of toxicity, *in vitro*^12–18^, *in vivo*^19–22^ and in clinical trials^23,24^ can be rationalised through the consideration of the lipid environment. DOX has been examined previously and shown to exhibit different behaviour when assessed in different environments within the cell membrane^28,29^. The success of membrane insertion observed from this study were in the rank order DOX<DAU~IDA<EPI for POPC and DMPC. The results obtained from this study, the success of penetration, are in reasonable correlation with the rank order IDA<DAU<EPI<DOX obtained from measurements of acute toxicity *in vitro* studies^12–18^ as well as measurements of their lipophilicity^30^. Additionally, it has been reported that DOX’s hydrophilic anthraquinone inserts into lipid bilayers spontaneously^31^. The interaction of DOX with membrane lipid bilayers was assessed with and without cholesterol present^29^, an important finding that emerged from these studies was that the presence of cholesterol induced a change in the structure of the membrane and this was solely due to its ordering effect and not through direct interaction with the drug. The simulations from this study revealed that the Doxorubicin cyclic group (dihydro-anthraquinone residue) was found perpendicular to the membrane when assessed without cholesterol. Additionally, a high partitioning of DOX was observed and this was explained in respect of its high ability to interact with the hydrocarbon lipid tails. These findings are in correlation with the results obtained from this study. Differences were observed for the orientational preferences of the Daunosamine sugar subunit of DOX compared to the other analogues, especially in the POPC lipid, where an angle bending was observed. The role of the Daunosamine sugar has been discussed previously where the interaction of Doxorubicin with a DOctPC bilayer was examined experimentally and complemented with molecular dynamics simulations^31^. From this study the DOctPC-mediated gateway afforded substantial solvation of doxorubicin (its Daunosamine sugar in particular) within the lipid bilayer core, which involved lipid headgroups lining the pore’s lumen and water molecules remaining associated with the sugar moiety. Additionally, the observed Doxorubicin channel formation was supported by the experimental findings.

The main conclusions which have emerged from this work are the significant differences in the behaviour of the Anthracyclines as a function of their chemical composition and structure and of the nature of the lipid bilayer. DOX’s behaviour is remarkably consistent, whether it is interacting with either POPC or DMPC, compared to the behaviour of the three other analogues: its speed of membrane insertion into the bilayer is at a much higher rate, its orientation relative to the z-axis of the membrane and finally its higher propensity to interact strongly with the hydrocarbon tails of the lipids. These three observations, we suggest provide an explanation for DOX’s high *in vitro* toxicity, and would therefore form a qualitative set of metrics suitable for assessing other potential therapeutic agents. The consistent rank order for the speed of membrane insertion DOX<DAU~IDA<EPI we believe can be correlated with the ligands rigidity as defined by the number of low frequency vibrational modes accessible to it, we believe this to be a further metric by which other ligands could be assessed. Finally, we would like to highlight the suitability of the use of unbiased MD to investigate and discriminate the behaviour of potential therapeutic agents as opposed to relatively high computational cost methods such as Potential of Mean Force (PMF) or Alchemical techniques such as Thermodynamic Integration (TI); in this study the computational cost of the nearly 100 simulations is still substantial, even with the speed advantage afforded by the use of the GPU accelerated MD. It would be beneficial if the number of repeats could be increased by an order of magnitude to increase the statistical confidence in the observations, however, performing a comparable number of repeats of for methods such as PMF or TI are simply intractable. We should, however, note that the use of unbiased MD is not suitable for studying rare events such as the complete movement of drug across a membrane.

## Methods

Molecular dynamics (MD) simulations have been performed for the four Anthracycline analogues in two different, POPC and DMPC, symmetric lipid bilayers. For the simulations, the four analogues were parameterised with the GAFF force field^31^. The electrostatic potential distribution was obtained with the Restrained Electrostatic Potential fit approach (RESP) through the multi-conformational fit approach^32^. The lipids were described with the LIPID14 force field^33^. The choice of these two lipids was intended to examine the behaviour of each of the analogues in different lipid environments (saturated DMPC and unsaturated POPC). The size of lipid bilayer was 128 lipids, the simulations were performed in an NPT ensemble and used the Berendsen barostat with a pressure relaxation time of 1 ps^34^. Periodic boundary conditions were set with the particle mesh Ewald method for long-range electrostatics interactions with a 10 Å cutoff for nonbonded interactions. Simulations employed the SHAKE constraints for bonds with hydrogen and used a 0.004 fs time step under the hydrogen mass repartitioning method. The simulation were run at 303 K. Hydrogen mass repartition was used in all the simulations^35^. The water model used to build the bilayers was TIP3P^36^. To the bilayer systems KCl of concentration of 0.1 M was added. For the POPC bilayer this resulted to the addition of 9 K^+^ and 9 CL^−^ ions for POPC and 7 K^+^ and 7 CL^−^ ions for DMPC. All the simulations were performed with the Amber16 program package^37^. Twelve repeats of 100 ns were performed for each of the four Anthracyclines in both lipids.

As the aim of this study was to monitor the early stages of the membrane insertion process, the four analogues were initially positioned 29 Angstroms above the centre of the bilayer placing them far enough away from the gel phase of the bilayer. The initial position of the Anthracyclines relative to the bilayer at the start of the simulations and the definition of the orientation relative to the z-direction are illustrated in Fig. 2. The lipids were solvated with experimental levels of hydration of the pure bilayer, the amount of water added to the lipid bilayer was 31 water molecules per lipid for POPC^38^ and 26.6 water molecules per lipid for DMPC^39^.

To address the different behaviour exhibited by DOX and EPI we performed *Ab Initio* DFT calculations to investigate their relative structures, energetics and vibrational properties. Geometry optimisations and vibrational frequency calculations were performed with the Gaussian 16^41^ program using the M062X functional^42^ and the cc-pvdz^43^ basis set. Standard program defaults for the optimisation and vibrational frequency calculations were used.

## Supplementary information

Supplementary information

Epirubicin 489 cm vibration

Epirubicin 551 cm vibration

Doxorubicin DMPC

Epirirubicin DMPC

Idarubicin DMPC

Daunorubicin in DMPC

Doxorubicin POPC

Epirubicin POPC

Idarubicin POPC

Daunorubicin POPC

Doxorubicin 209 cm vibration

Doxorubicin 231 cm vibration

Doxorubicin 333 cm vibration

Doxorubicin 354 cm vibration

Doxorubicin 367 cm vibration

Doxorubicin 472 cm vibration

Epirubicin 281 cm vibration

Epirubicin 287 cm vibration

Epirubicin 292 cm vibration

Epirubicin 353 cm vibration

Epirubicin 347 cm vibration

Epirubicin 302 cm vibration

Epirubicin 368 cm vibration

Epirubicin 422 cm vibration

Epirubicin 453 cm vibration

Epirubicin 468 cm vibration
