# Supplementary material for: A computational study of Anthracyclines interacting with lipid bilayers: Correlation of membrane insertion rates, orientation effects and localisation with cytotoxicity
Source: Sci Rep. 2019 Feb 15;9:2155. doi: 10.1038/s41598-019-39411-y (PMC6377671; doi:10.1038/s41598-019-39411-y)
Supplement: Supplementary file 1 — Supplementary information [file 41598_2019_39411_MOESM1_ESM.pdf]

A computational study of Anthracyclines interacting with lipid bilayers: Correlation of membrane insertion rates, orientation effects and localisation with cytotoxicity.

D. Toroz[a] and I.R. Gould[a*]

[a] *Department of Chemistry, Imperial College London,* SW7 2AZ, UK

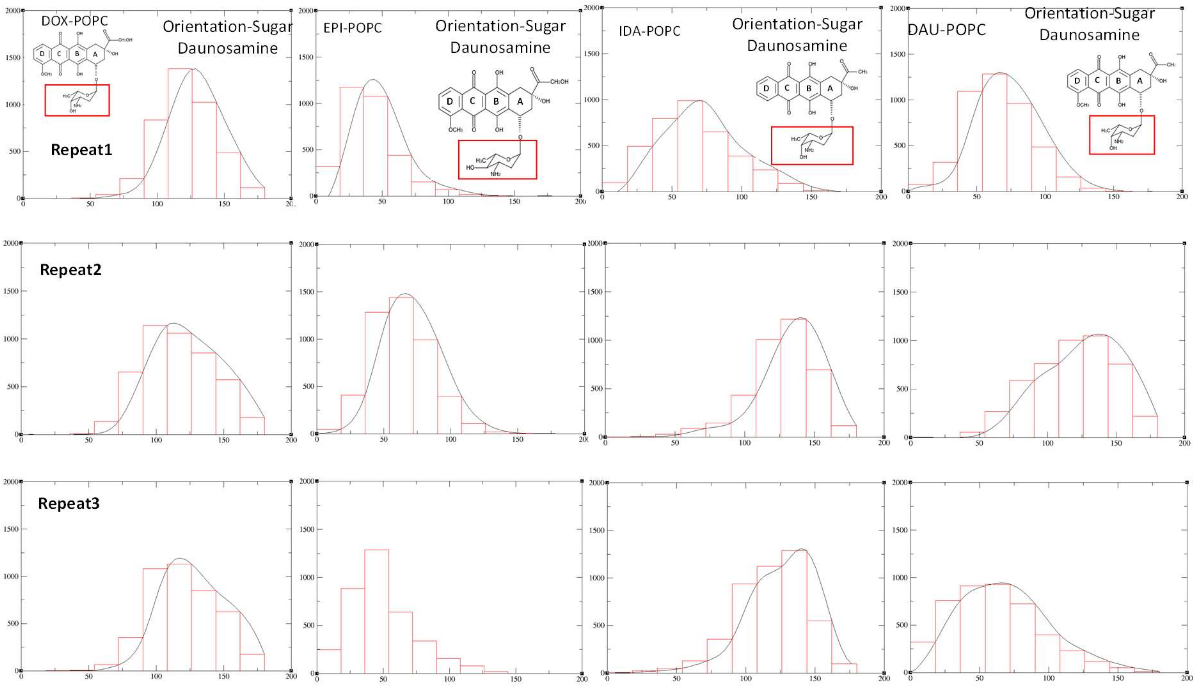

Figure 1Sa Orientation preferences (angle distribution of the Daunosamine subunit of the Anthracyclines in POPC).

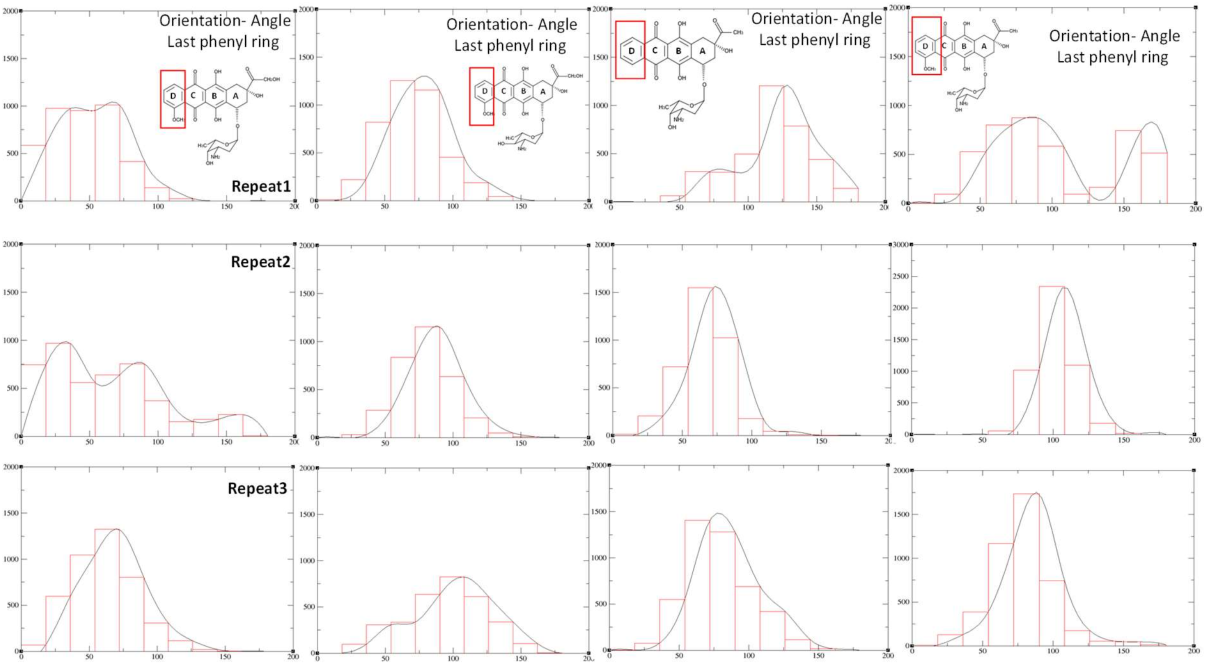

Figure 1Sb Orientation preferences (angle distribution of the last phenyl ring of the anthraquinone subunit of the Anthracyclines in POPC).

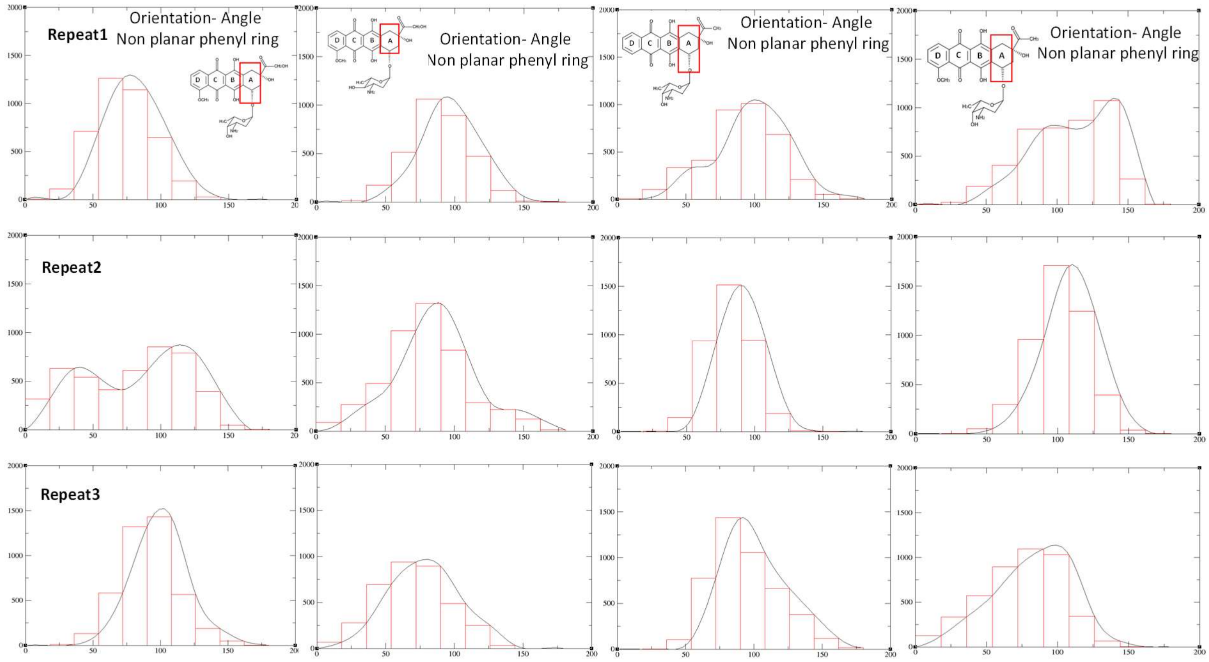

Figure 1Sc Orientation preferences (angle distribution of the non-planar aromatic ring of the anthraquinone subunit of the Anthracyclines in POPC).

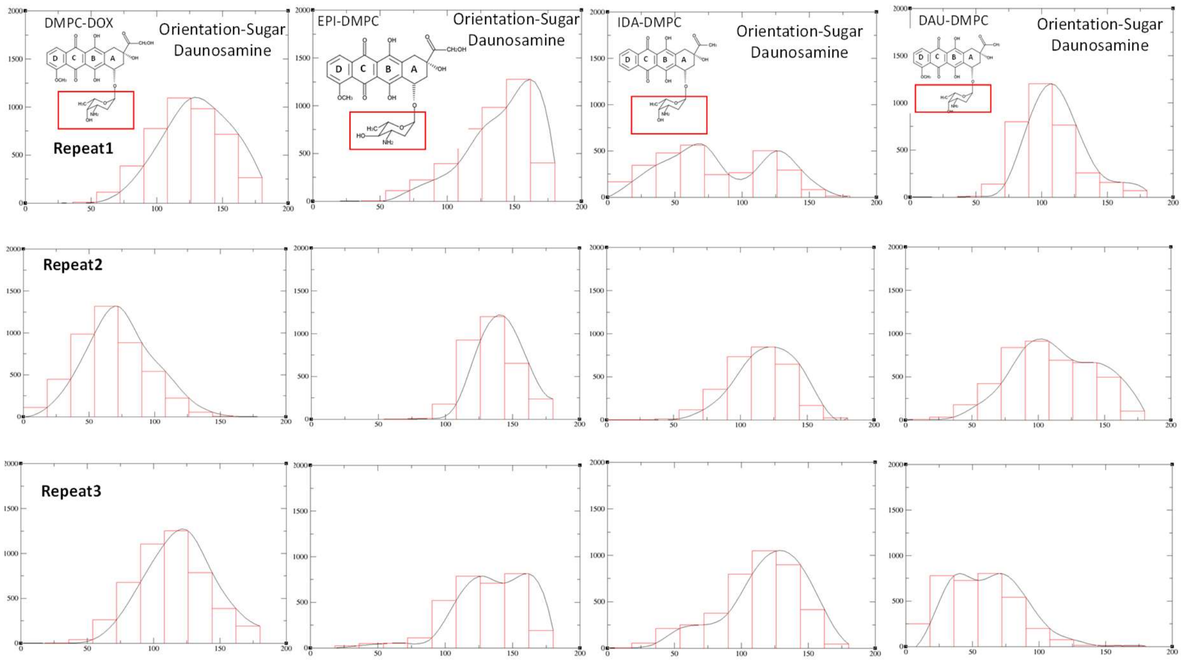

Figure 2Sa Orientation preferences (angle distribution of the Daunosamine subunit of the Anthracyclines in DMPC).

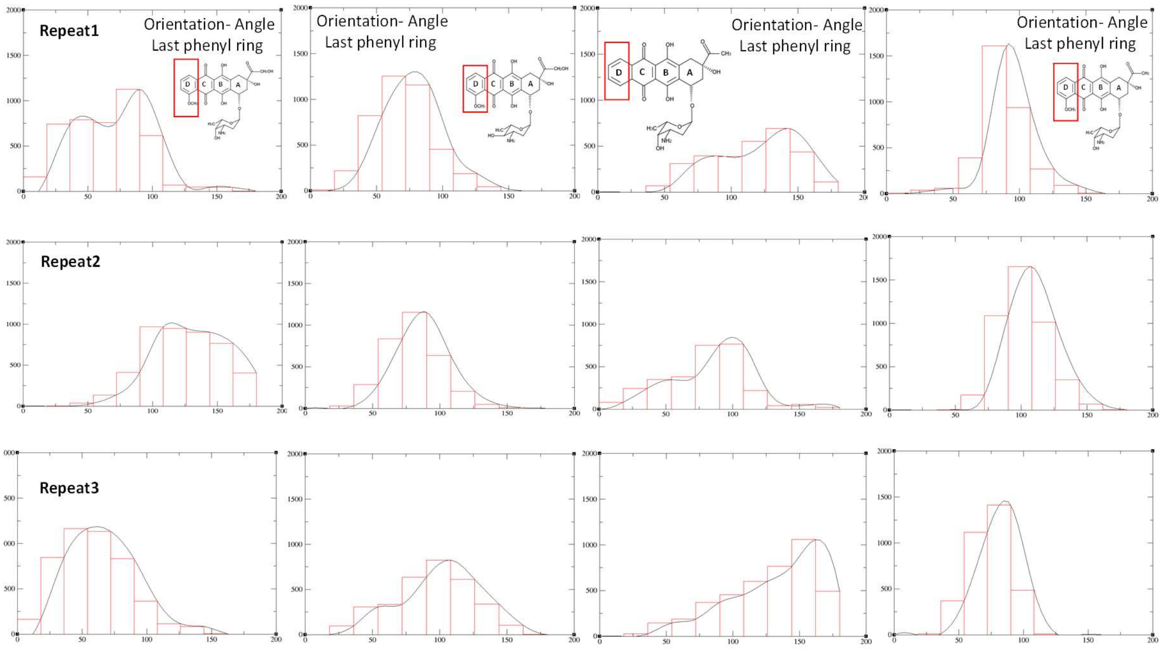

Figure 2Sb Orientation preferences (angle distribution of the last phenyl ring of the anthraquinone subunit of the Anthracyclines in DMPC).

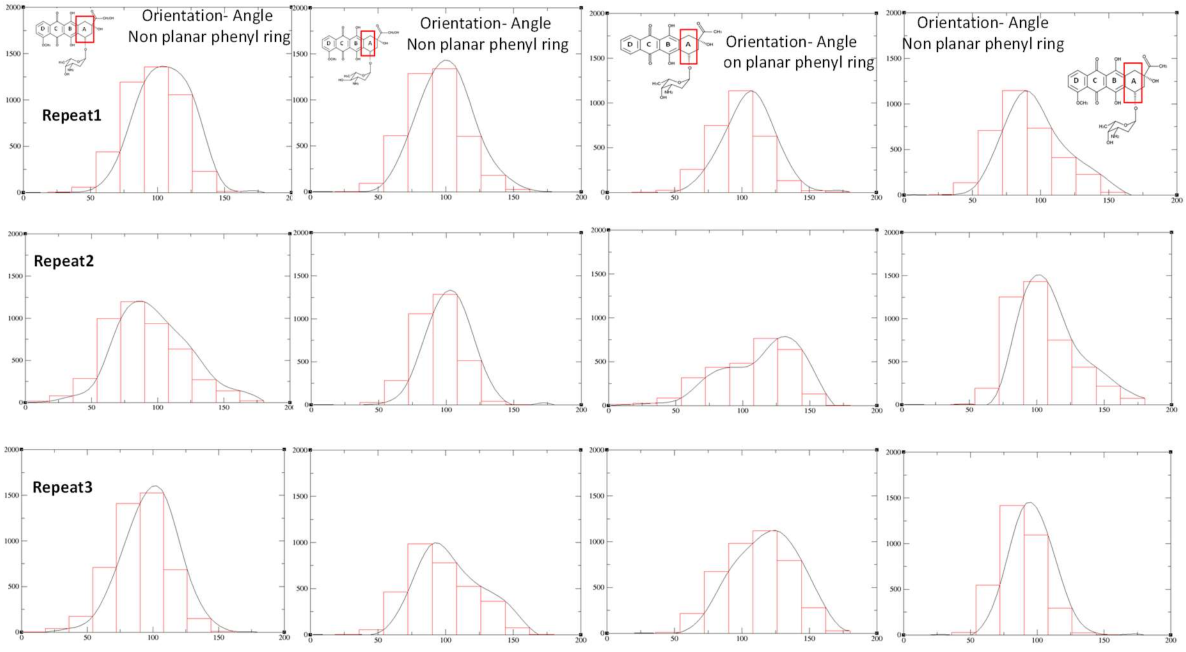

Figure 2Sc Orientation preferences (angle distribution of the non-planar aromatic ring of the anthraquinone subunit of the Anthracyclines in DMPC).

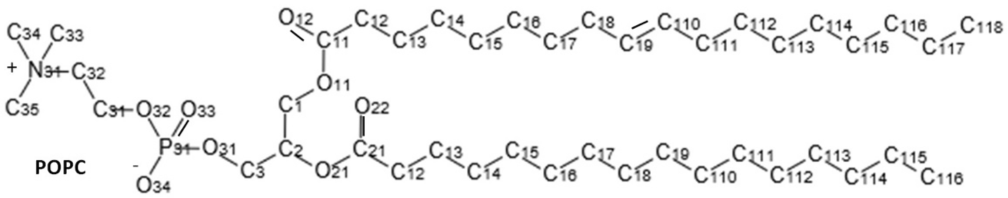

POPC

Bins selected for creating the histograms:**(1-6)**:C_{35}-C_{33}-N_{31}-C_{34}-C_{32}-C_{31} **(6-11)**:O_{32}-O_{34}-P_{31}-O_{33}-O_{31}
POPC **(11-16)**:C_3-C_2-C_1-O_{11}-O_{21} **(16-21)**: C_{21}-O_{22}-C_{11}-O_{12}-C_{12}
(21-26):C_{13}-C_{14}-C_{15}-C_{16}-C_{17} **(26-31)**: C_{18}-C_{19}-C_{110}-C_{111}-C_{112}
(31-36):C_{113}-C_{114}-C_{115}-C_{116}-C_{117} **(36-41)**:C_{118}

Bins selected for creating the histograms:**(1-6)**:C_{35}-C_{33}-N_{31}-C_{34}-C_{32}-C_{31} **(6-11)**:O_{32}-O_{34}-P_{31}-O_{33}-O_{31}
DMPC **(11-16)**:C_3-C_2-C_1-O_{11}-$O_2$1 **(16-21)**: C_{21}-O_{22}-C_{11}-O_{12}-C_{12}
(21-26):C_{13}-C_{14}-C_{15}-C_{16}-C_{17} **(26-31)**: C_{18}-C_{19}-C_{110}-C_{111}-C_{112}
(31-36): C_{113}-C_{114}

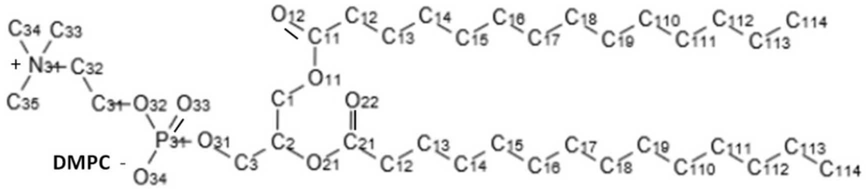

DMPC

Figure 3S. Nomenclature and definition of the bins selected to generate the histograms to illustrate the preferences of interactions of Anthracyclines with different components of lipid bilayers.

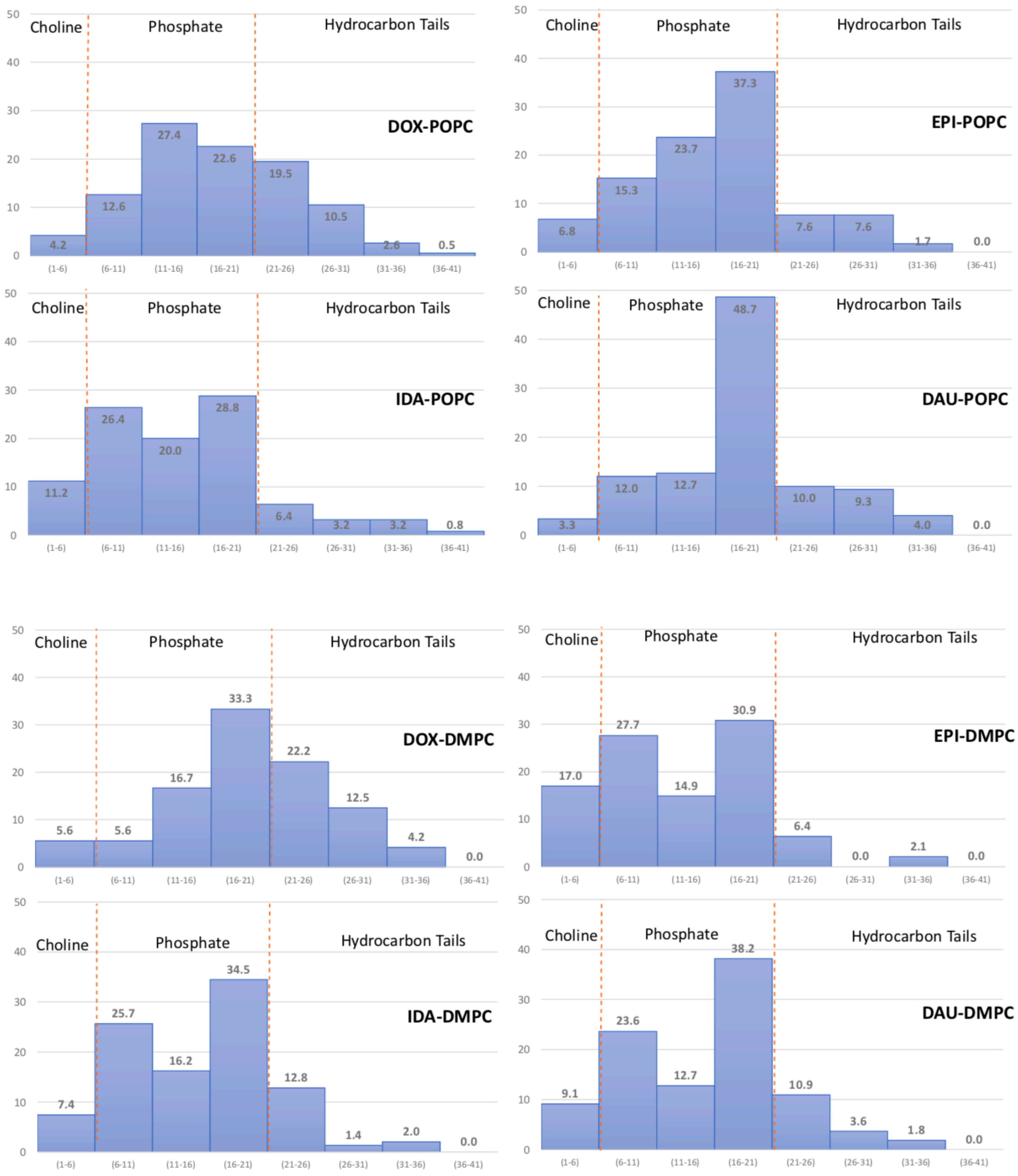

Figure 4S Preference of interactions between the four Anthracyclines during the simulation with the different regions of the two lipids **(first 60 ns)** The x-axis represents the bin of the histograms whereas the y-axis represents as a percentage the occurrence of each of the interactions of Anthracyclines with the specific region of the lipid components.

Figure 5S Molecular Orbital's are presented such that Dox is always above Epi below for each MO

Mo = 135

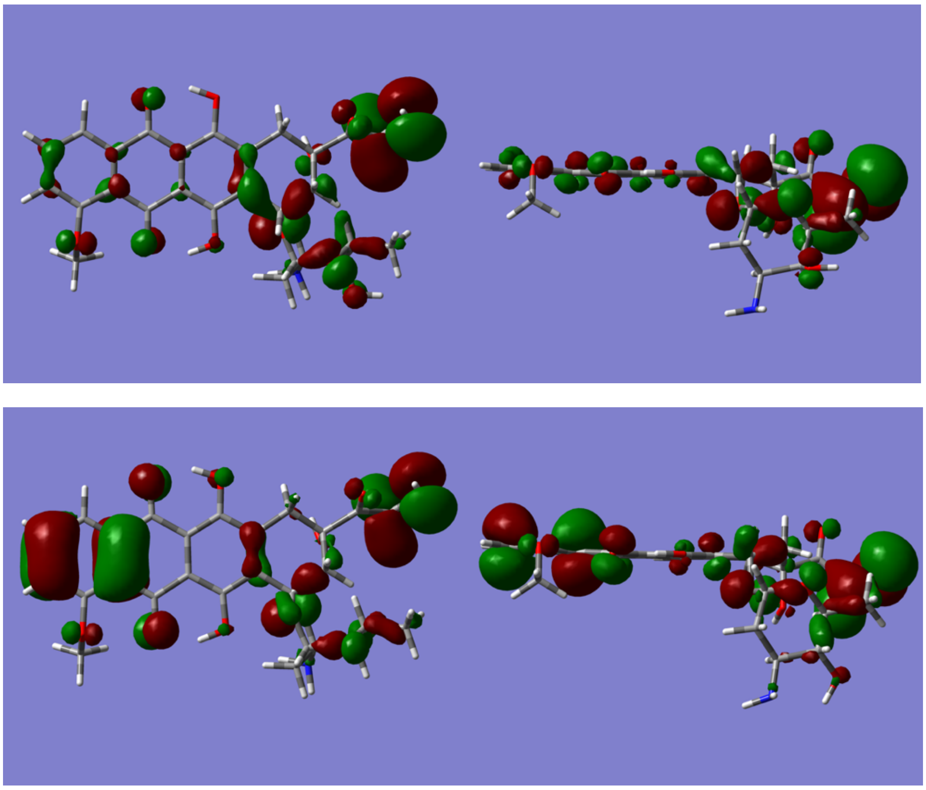

MO=136

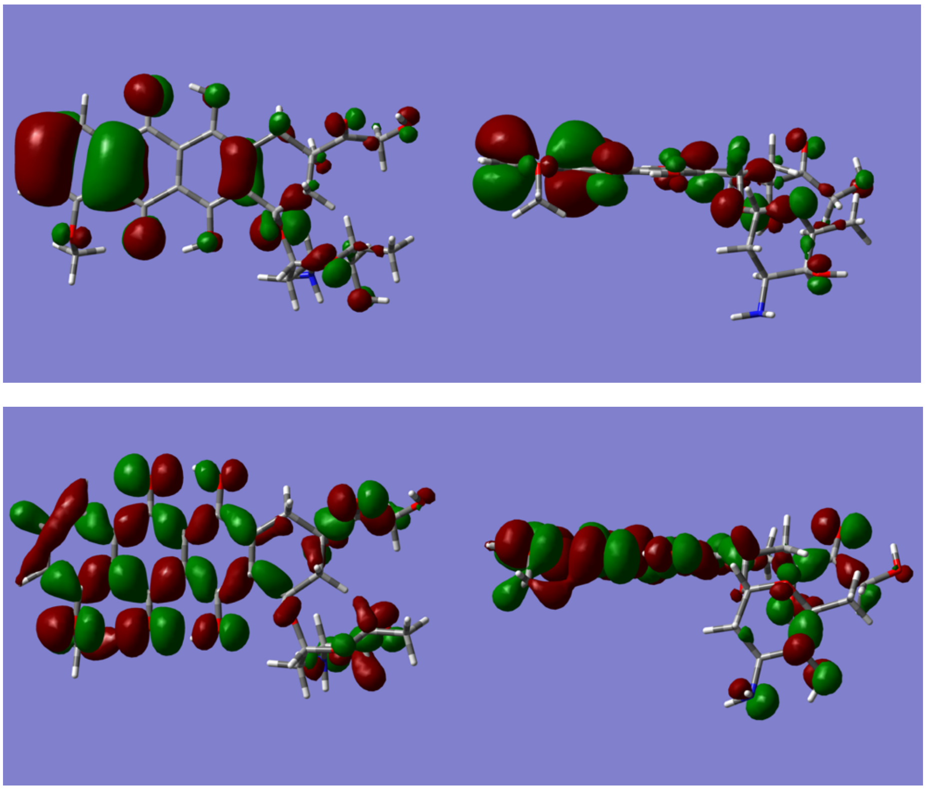

Mo = 137

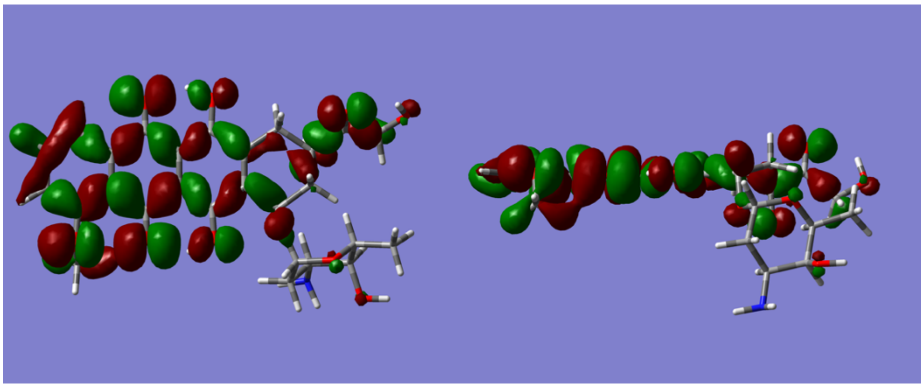

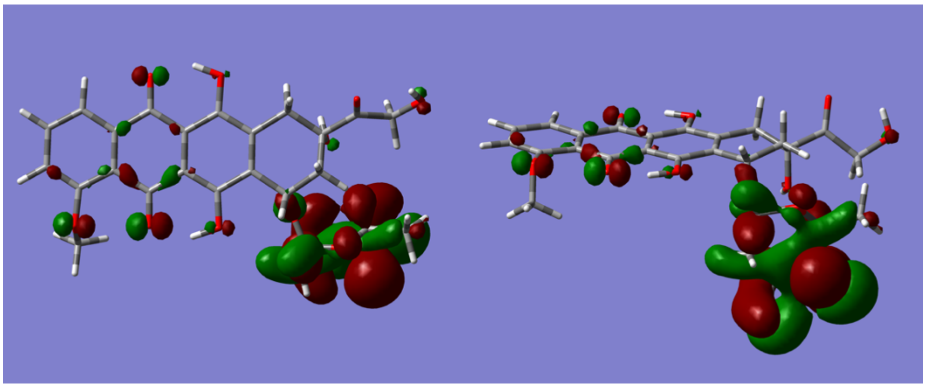

MO=138

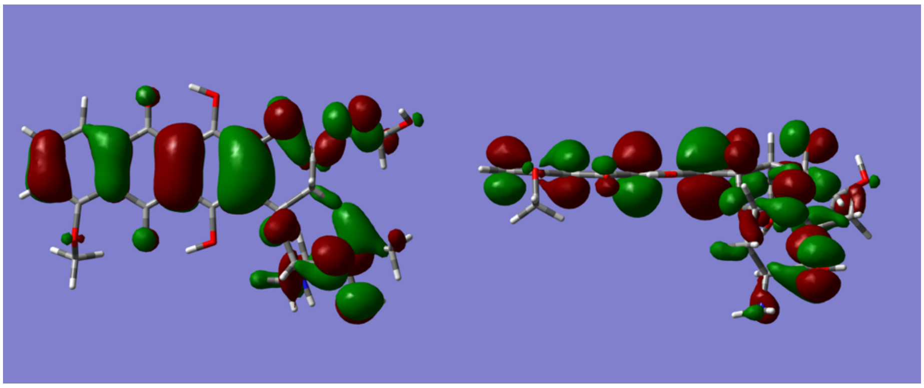

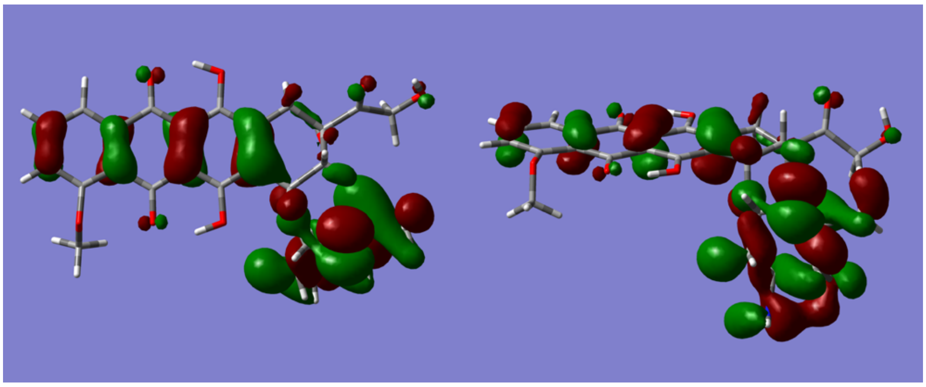

MO=139

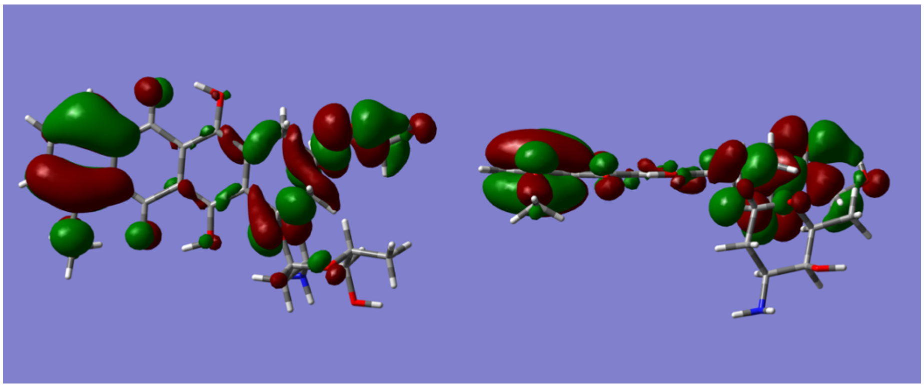

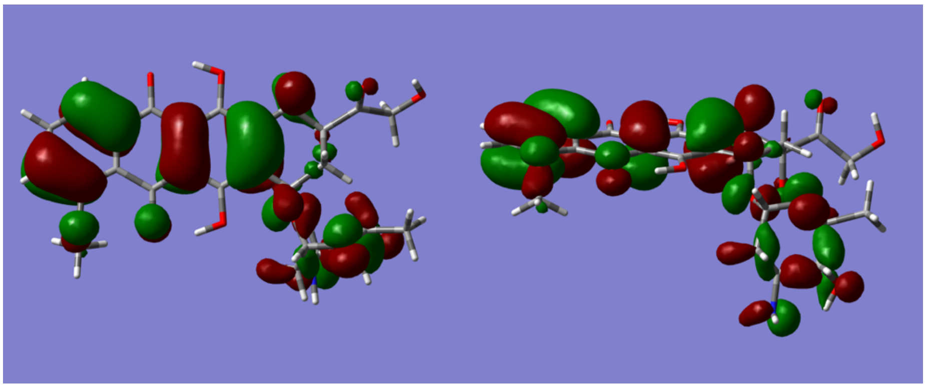

MO=140

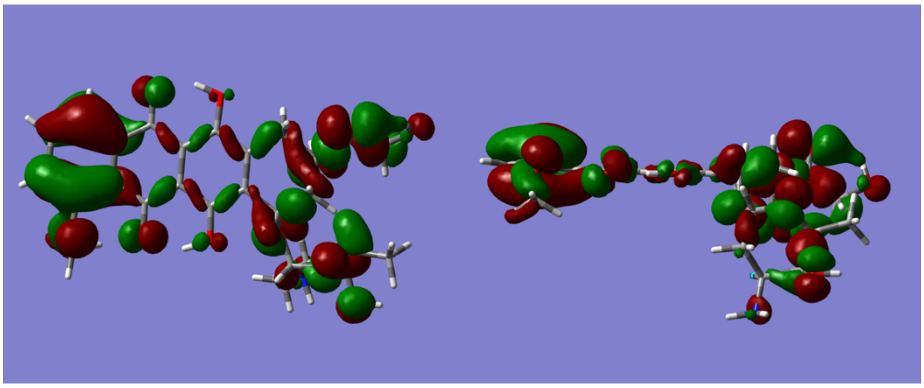

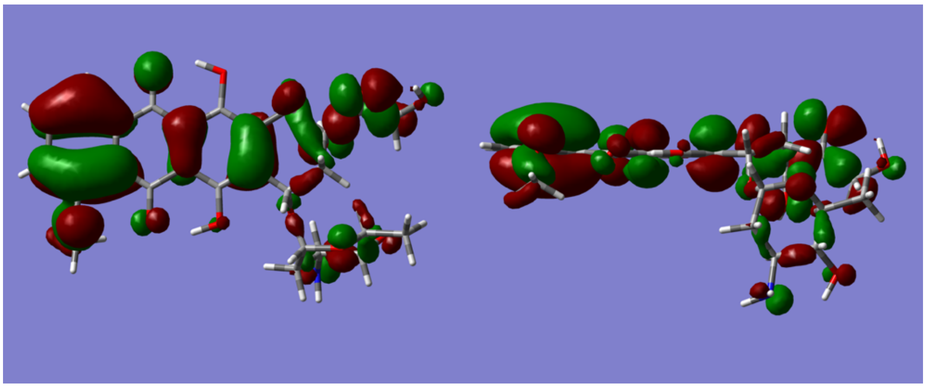

MO=141

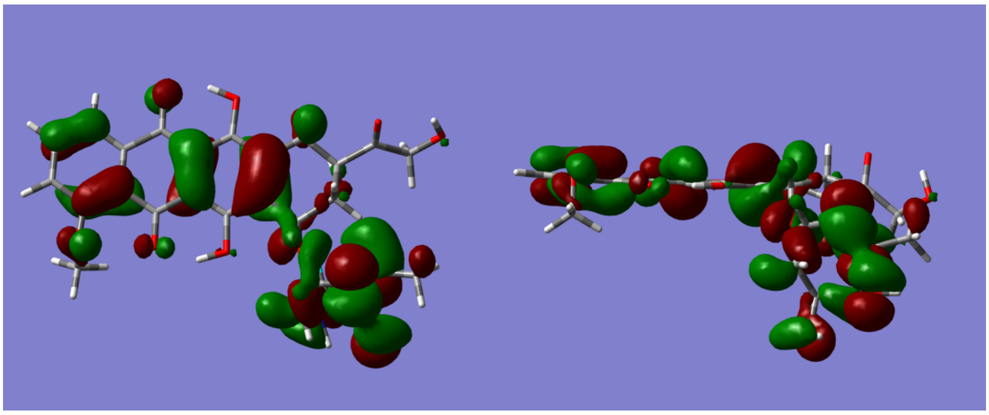

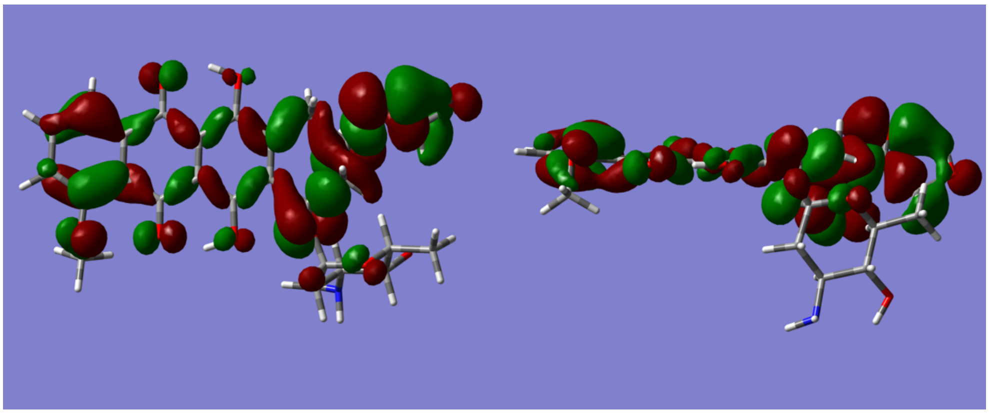

MO=142

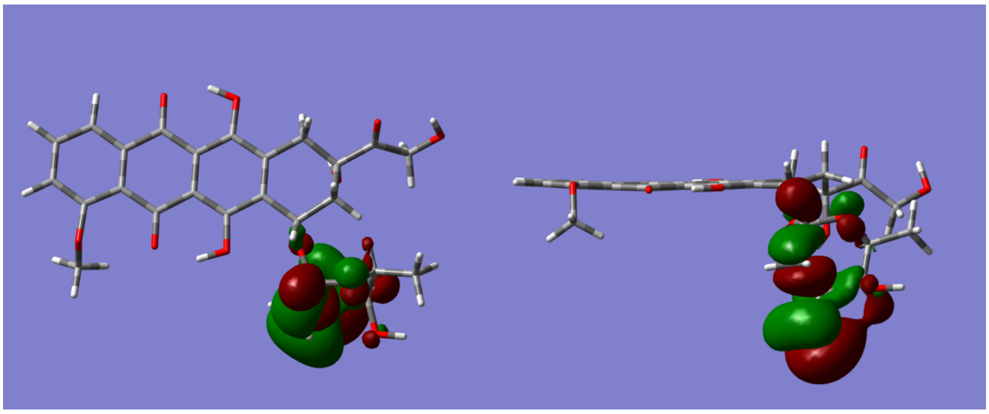

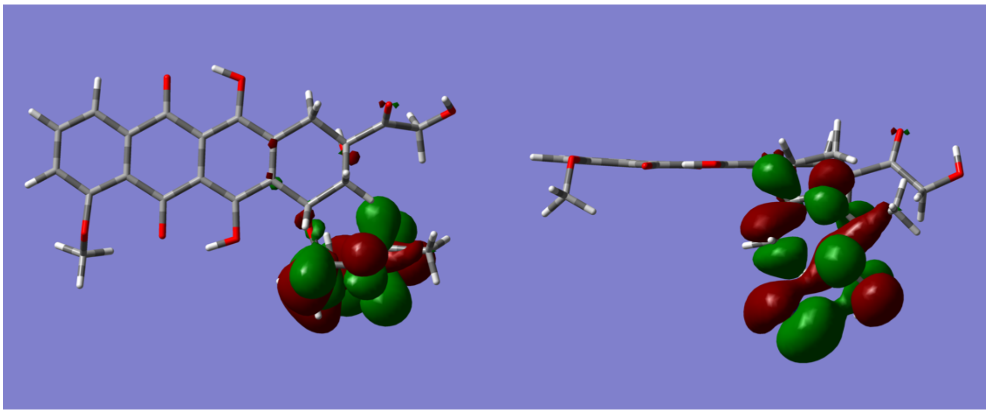

MO=143

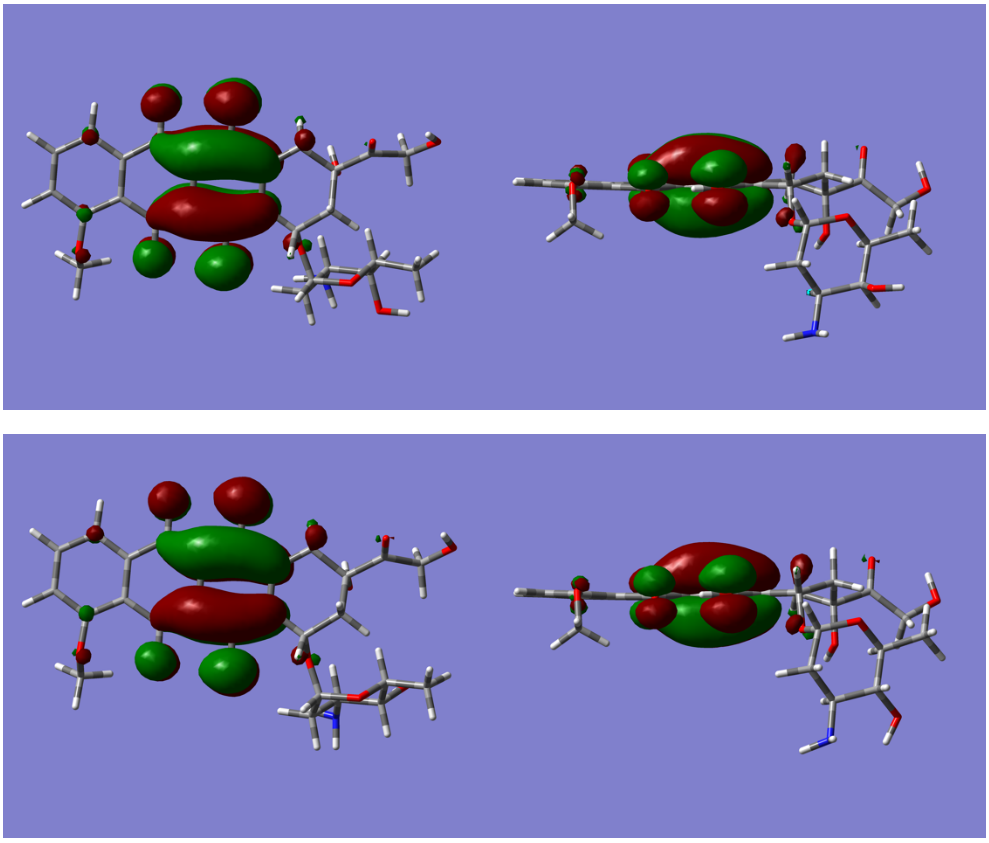

MO=144

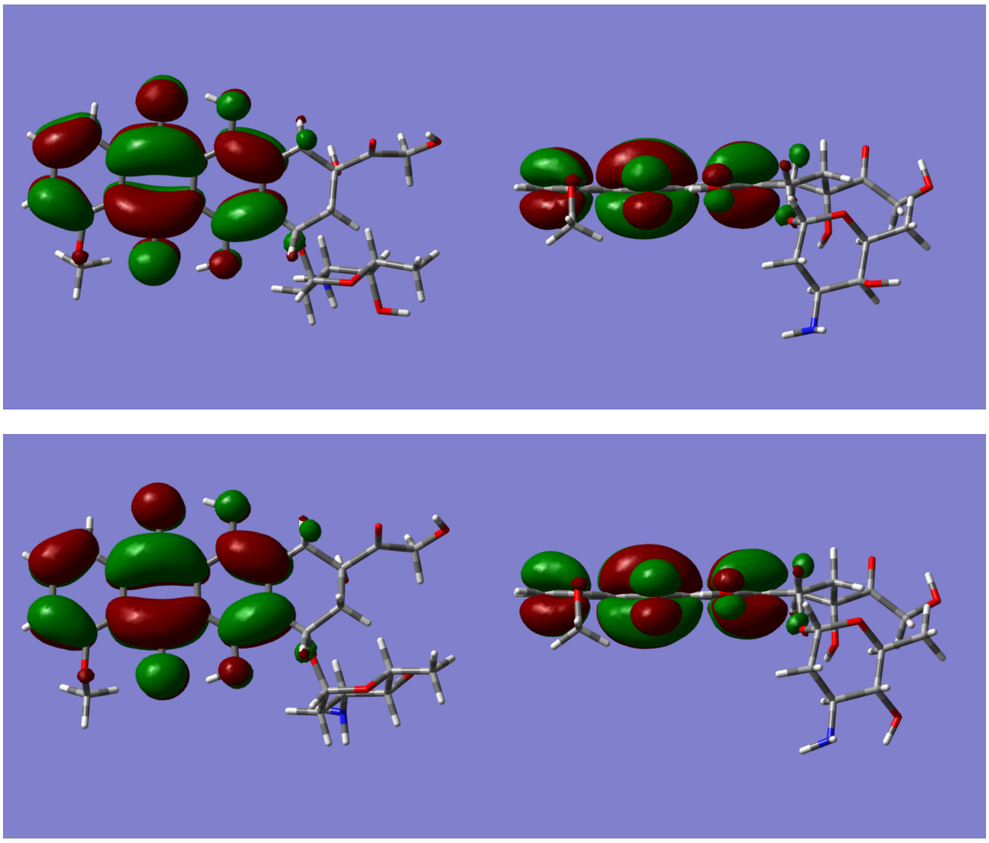

MO=145

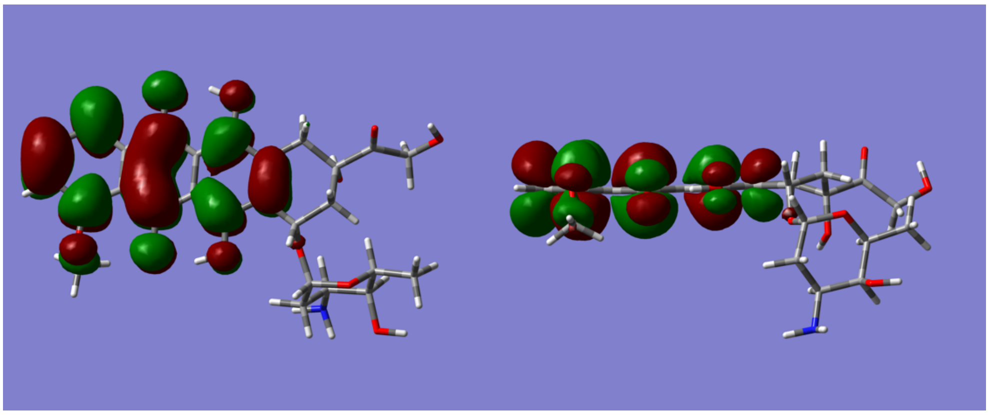

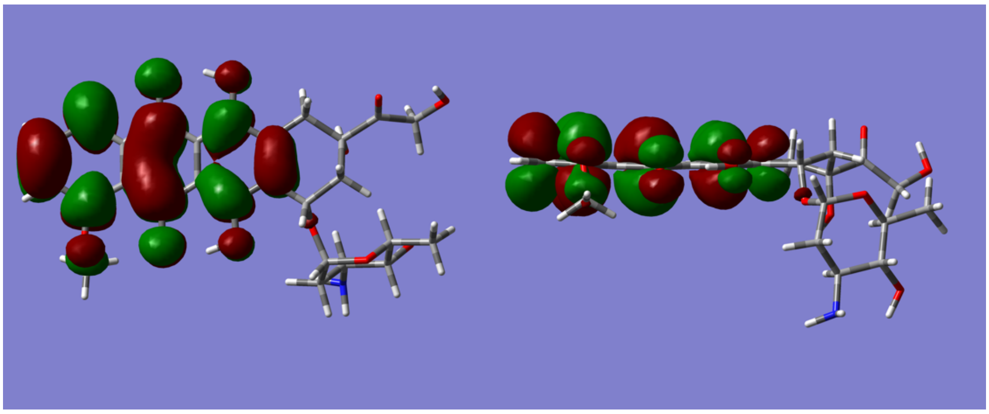

MO=146

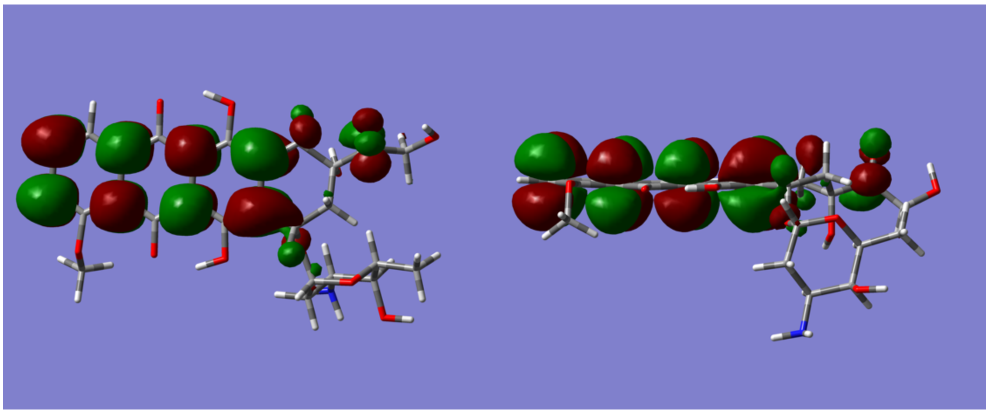

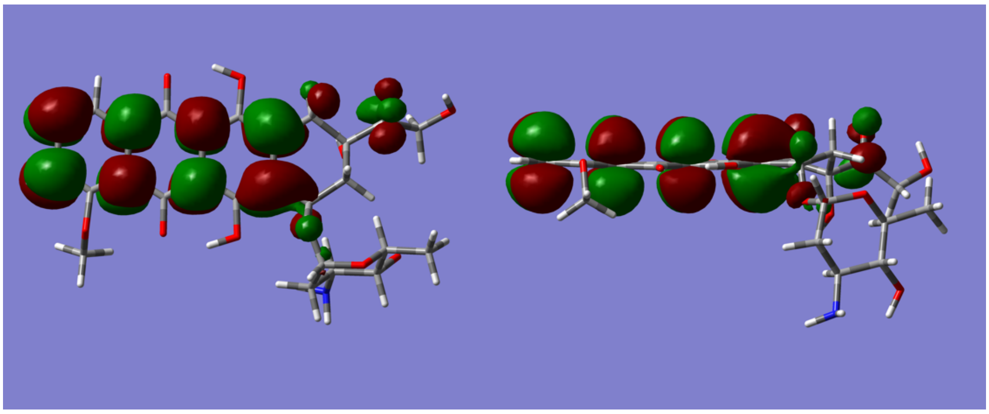

MO=147

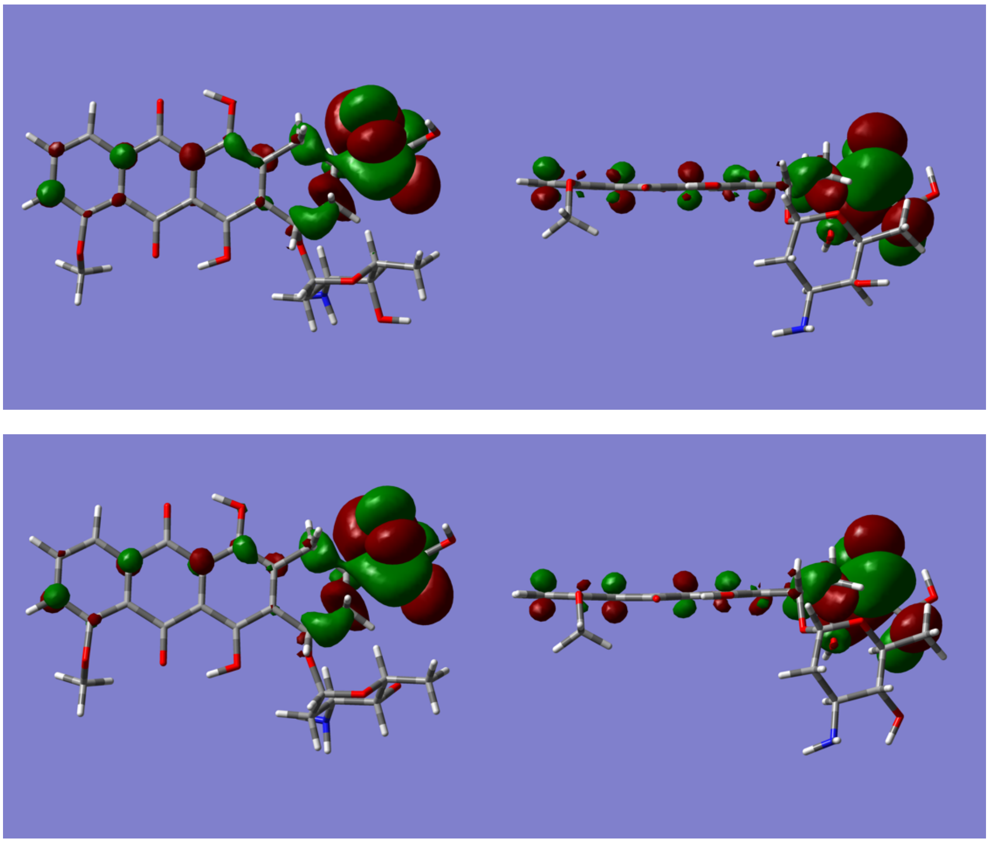

MO=148

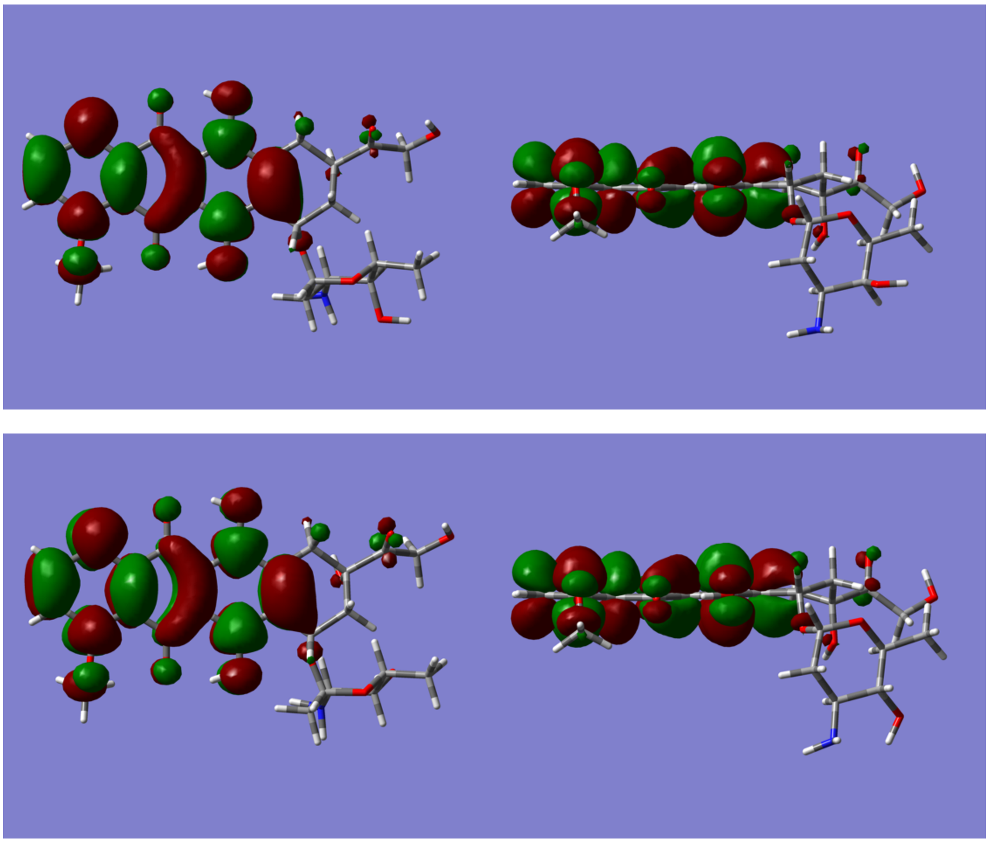

MO=149

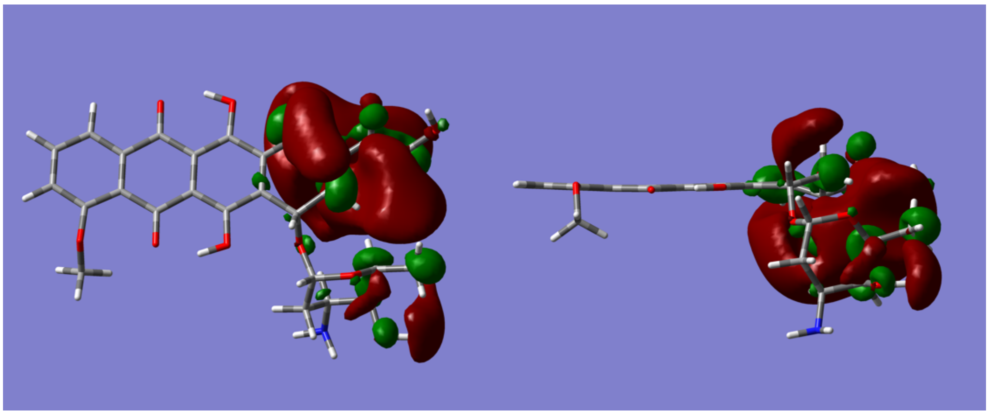

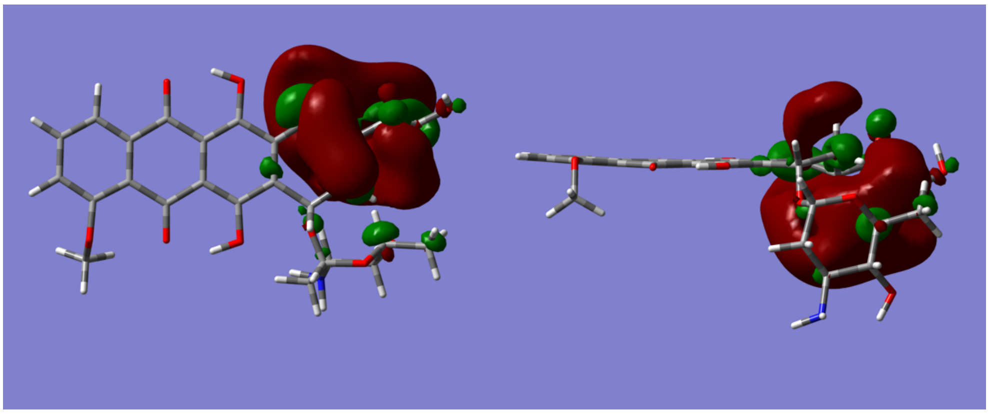

MO=150

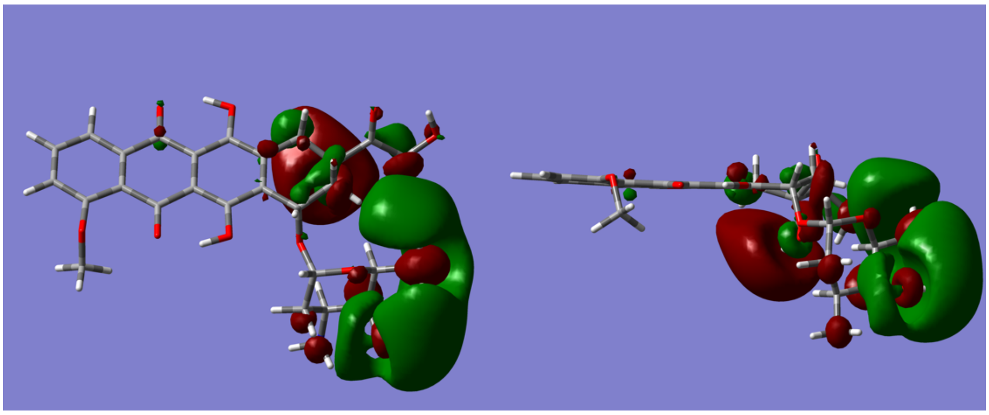

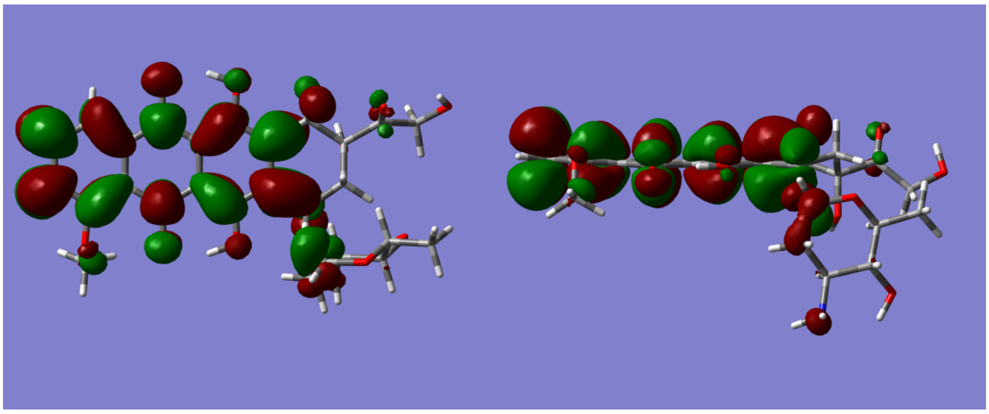

MO=151

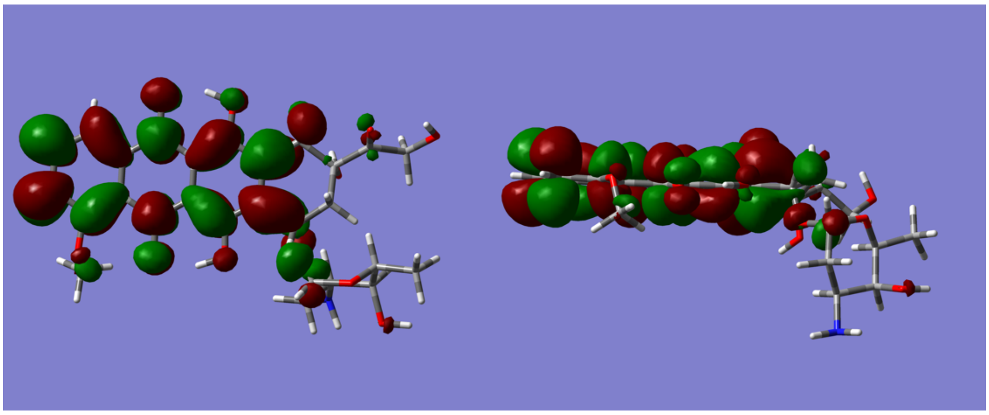

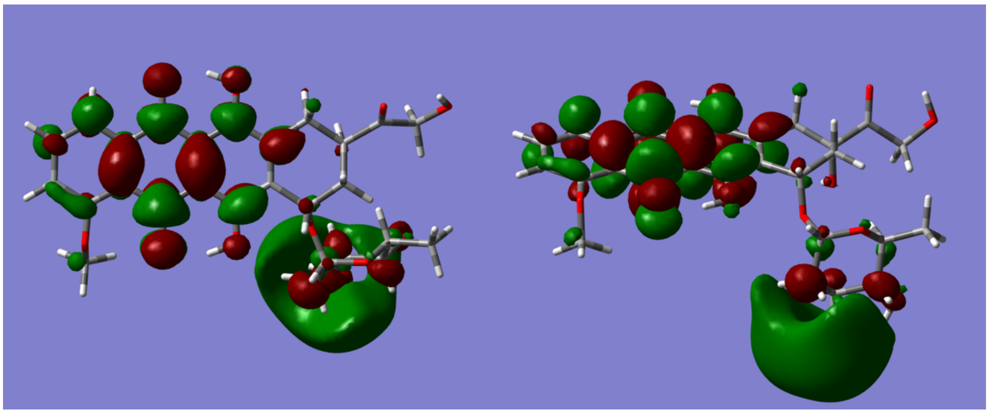

MO=152

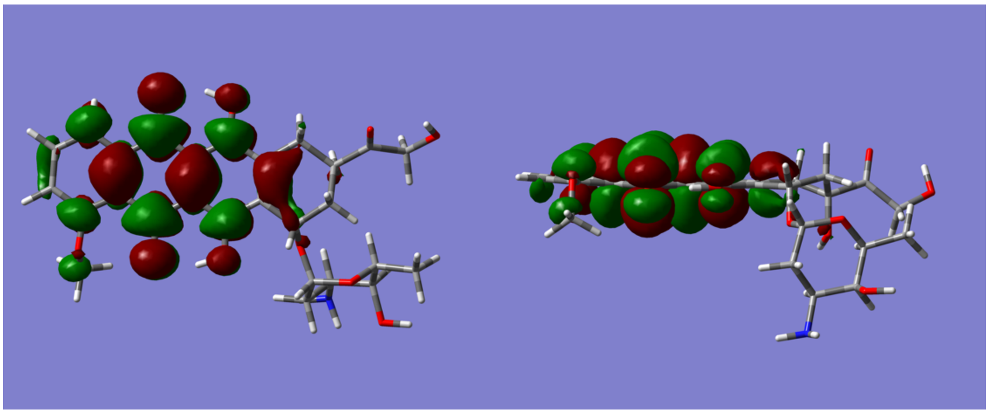

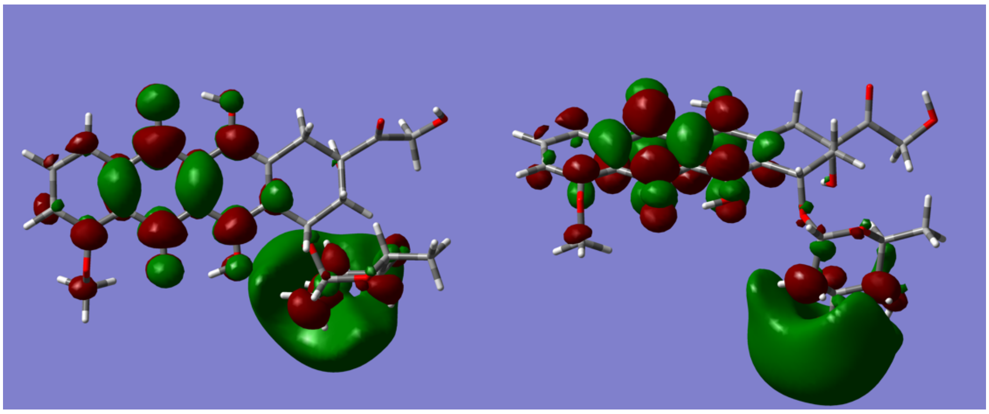

Figure 6S. Full calculated Infra-Red spectra of a) DOX, b) EPI, c) Ida and d) DAU

a)

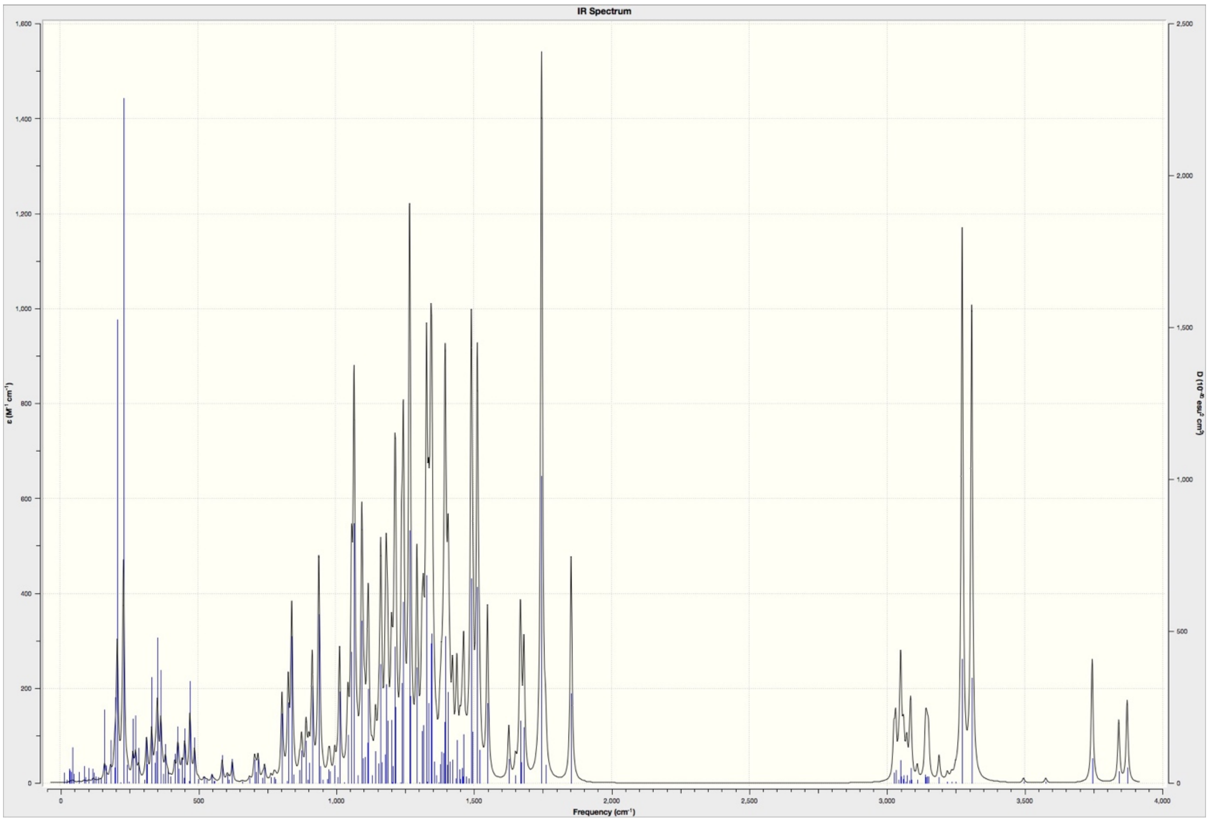

b)

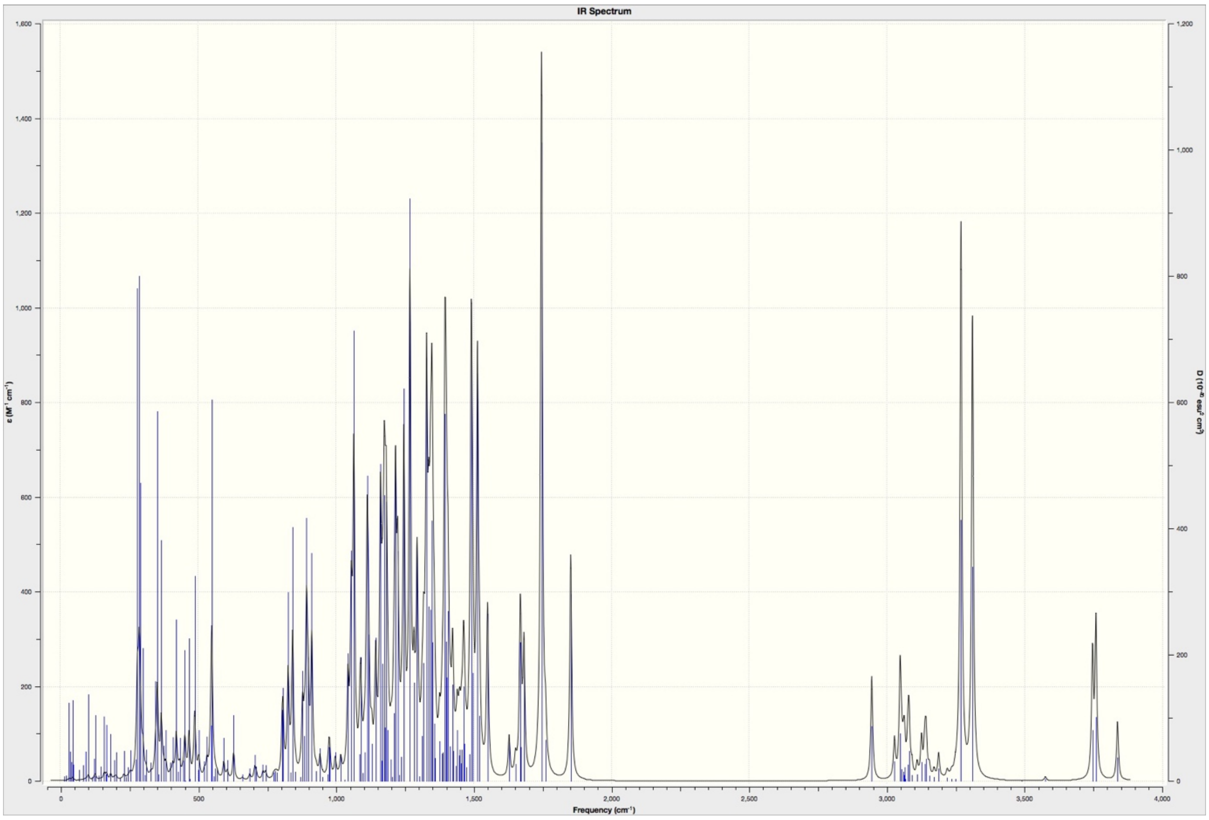

c)

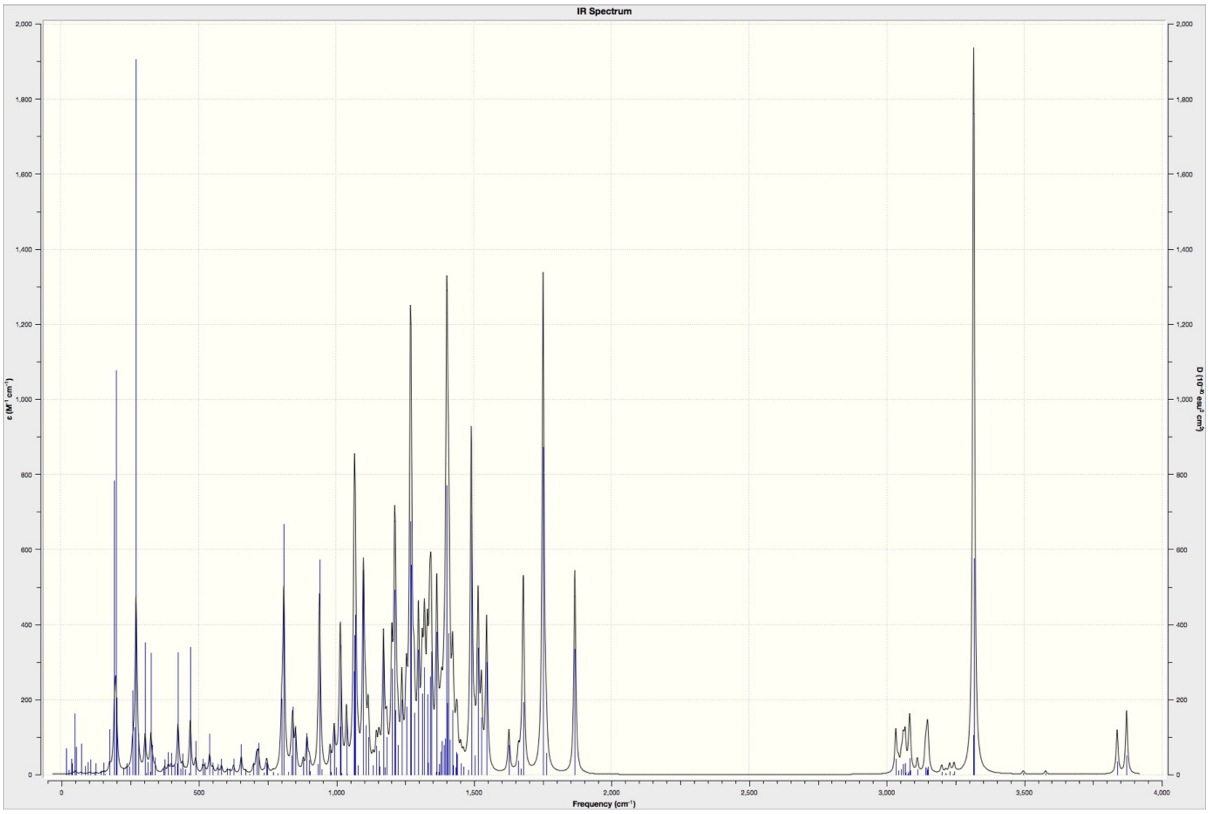
IR Spectrum
ε (M⁻¹ cm⁻¹)
D (10⁻⁴⁰ esu² cm²)
Frequency (cm⁻¹)

d)

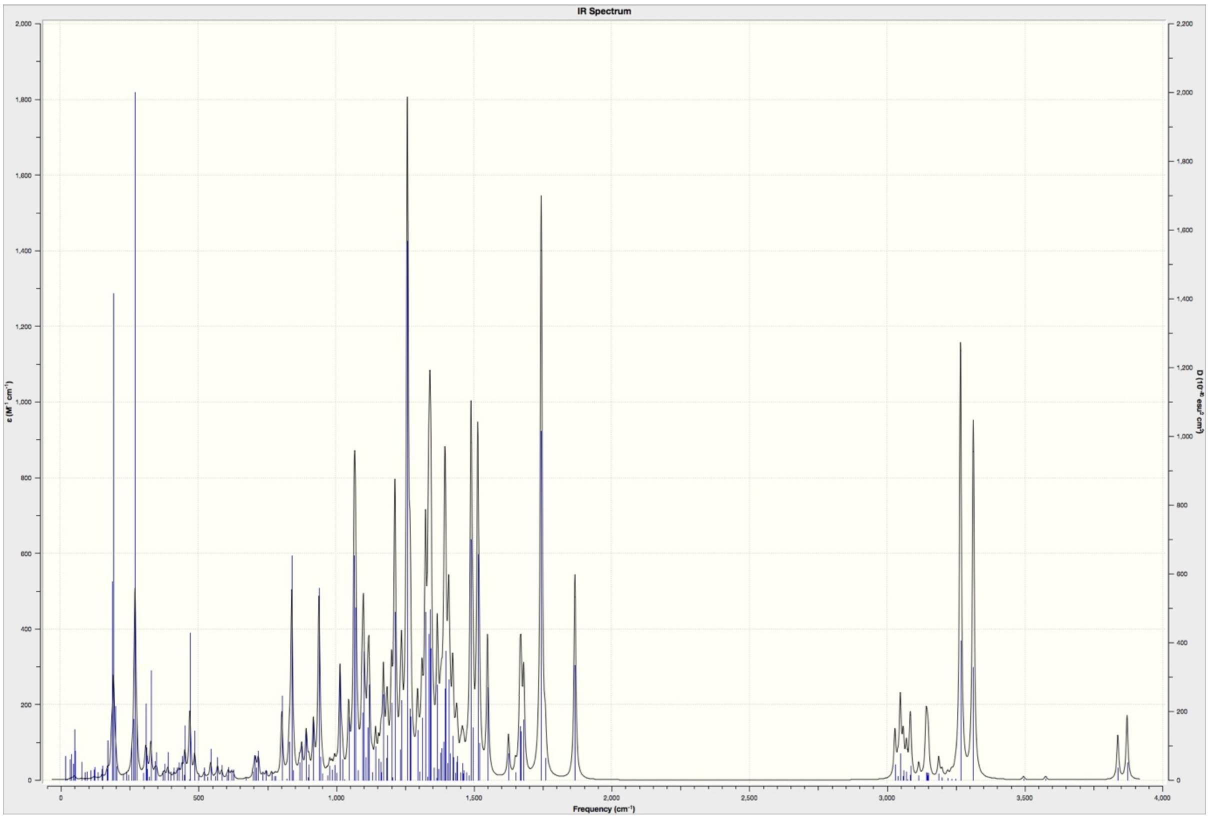
IR Spectrum
ε (M⁻¹ cm⁻¹)
D (10⁻⁴⁰ esu² cm²)
Frequency (cm⁻¹)
